# Agricultural Waste-Derived Biopolymers for Sustainable Food Packaging: Challenges and Future Prospects

**DOI:** 10.3390/polym17141897

**Published:** 2025-07-09

**Authors:** Thivya Selvam, Nor Mas Mira Abd Rahman, Fabrizio Olivito, Zul Ilham, Rahayu Ahmad, Wan Abd Al Qadr Imad Wan-Mohtar

**Affiliations:** 1 Department of Chemistry, Faculty of Science, Universiti Malaya, Kuala Lumpur 50603, Malaysia; thivyaselvam1998@gmail.com; 2 Department of Environmental Engineering, University of Calabria, Via P. Bucci, 87036 Arcavacata di Rende, CS, Italy; 3 Environmental Science and Management Program, Institute of Biological Sciences, Faculty of Science, Universiti Malaya, Kuala Lumpur 50603, Malaysia; ilham@um.edu.my; 4 Halal Action Laboratory, Kolej PERMATA Insan, Universiti Sains Islam Malaysia, Bandar Baru Nilai, Nilai 71800, Malaysia; rahayu@usim.edu.my; 5 Functional Omics and Bioprocess Development Laboratory, Institute of Biological Sciences, Faculty of Science, Universiti Malaya, Kuala Lumpur 50603, Malaysia; qadyr@um.edu.my

**Keywords:** biopolymer, agricultural waste, food packaging, sustainability, circular economy

## Abstract

The widespread use of conventional plastic in food packaging has raised serious environmental issues due to its persistence and poor biodegradability. With growing concerns over plastic pollution and its long-term ecological impact, researchers are increasingly turning to natural, renewable sources for sustainable alternatives. Agricultural waste, often discarded in large quantities, offers a valuable resource for producing biodegradable polymers. This review discusses the environmental burden caused by traditional plastics and explores how agricultural residues such as rice husks, corn cobs, and fruit peels can be converted into eco-friendly packaging materials. Various types of biopolymers sourced from agricultural waste, including cellulose, starch, plant and animal-based proteins, polyhydroxyalkanoates (PHA), and polylactic acid (PLA), are examined for their properties, benefits, and limitations in food packaging applications. Each material presents unique characteristics in terms of biodegradability, mechanical strength, and barrier performance. While significant progress has been made, several challenges remain, including cost-effective production, material performance, and compliance with food safety regulations. Looking ahead, innovations in material processing, waste management integration, and biopolymer formulation could pave the way for widespread adoption. This review aims to provide a comprehensive overview of current developments and future directions in the use of agricultural waste for sustainable packaging solutions, comparing their biodegradability and performance to conventional plastics.

## 1. Introduction

The excessive use of petroleum-based plastics in food packaging has led to severe environmental pollution and health concerns. With global plastic waste projected to increase, there is an urgent need for biodegradable and sustainable alternatives. Agricultural waste offers a promising source for biopolymer production, as it is abundant, renewable, and often discarded as a byproduct. This review explores different types of biopolymers derived from agricultural waste, their functional properties, processing techniques, and potential applications in sustainable food packaging.

### 1.1. Background on Plastic Waste and Its Environmental Impact

Plastics have become an indispensable part of modern life, revolutionizing industries and transforming the way we manufacture, store, and transport goods. Their widespread use can be attributed to their advantageous properties, such as lightweight, high durability, chemical resistance, and low cost of production [1]. From consumer packaging and automotive parts to electronics, textiles, and medical devices, plastics have enabled technological and industrial advancements across virtually all sectors. However, the very qualities that make plastics so useful, especially their resistance to degradation, are the same features that have contributed to a growing global environmental crisis: plastic pollution [2,3].

The production of plastics has increased dramatically since their large-scale introduction in the mid-20th century. According to a report by Plastics Europe (2020), global plastic production reached 367 million metric tons in 2020, a sharp increase from just 2 million metric tons in 1950 [4]. A substantial portion of this plastic, approximately 40%, is used for packaging purposes, most of which is intended for single use [4]. After a short period of utility, these materials are often discarded, leading to the generation of massive amounts of waste. Unfortunately, only a small percentage of plastic waste is effectively recycled—estimated at less than 10% globally, with the rest ending up in landfills, incineration plants, or natural environments [5,6].

The environmental consequences of this mismanaged plastic waste are profound and far-reaching. Plastics can take anywhere from 20 to 500 years to decompose, depending on the type and environmental conditions [6,7]. Over this prolonged degradation period, plastics break down into smaller fragments known as microplastics (particles less than 5 mm in size). These microplastics are now found in nearly every corner of the planet, from the deepest parts of the ocean to remote mountain peaks and polar ice caps. Their persistence poses a long-term threat to ecosystems and public health [6,7,8].

#### 1.1.1. Marine Pollution and Biodiversity Threat

One of the most visible and devastating impacts of plastic waste is its accumulation in the marine environment. According to the United Nations Environment Programme (UNEP), an estimated 8 to 11 million tons of plastic waste enter the oceans each year, forming vast patches of floating debris, such as the infamous Great Pacific Garbage Patch. This pollution affects over 800 marine species, including fish, seabirds, turtles, and marine mammals, many of which ingest plastic debris or become entangled in it. Sea turtles, for instance, often mistake plastic bags for jellyfish, while seabirds have been found with stomachs full of plastic fragments, leading to starvation and death [9,10,11].

Ingestion of plastic not only causes physical blockages and internal injuries in marine animals but also exposes them to toxic chemicals [12]. Additives used in plastic production, such as phthalates, bisphenol A (BPA), and flame retardants, can leach into marine ecosystems, disrupting hormone functions and reproductive systems in aquatic species. Furthermore, plastics act as vectors for persistent organic pollutants (POPs), absorbing toxins from the surrounding environment and transporting them through the food web [13,14].

Microplastics have also been detected in the guts of zooplankton, fish, and shellfish—organisms that form the base of the aquatic food chain. As a result, these contaminants can bioaccumulate and biomagnify, eventually reaching humans through seafood consumption. Recent studies have found microplastics in human blood, lungs, placenta, and breast milk, raising concerns about long-term health implications, including inflammation, oxidative stress, and potential carcinogenic effects [15,16,17].

#### 1.1.2. Plastic Waste in Terrestrial and Atmospheric Systems

While marine ecosystems have received significant attention, terrestrial environments are equally affected by plastic pollution. Agricultural lands, for instance, are increasingly contaminated with plastic mulch, irrigation systems, and packaging waste [18]. As plastics degrade in the soil, they affect soil structure, water retention, and microbial communities, which can in turn impact crop productivity and food security [19].

Plastics also contribute to atmospheric pollution. During degradation, especially under sunlight (photodegradation), plastics release greenhouse gases such as methane and ethylene, contributing to climate change. Incineration of plastic waste, although used as a method of volume reduction and energy recovery, releases harmful gases including dioxins, furans, and heavy metals, which pose serious risks to both human health and the environment [20,21]. Moreover, the production and life cycle of plastic are highly carbon-intensive. From fossil fuel extraction to polymerization and transportation, every stage emits greenhouse gases, adding to the global carbon footprint [20,22]. It is estimated that plastic production accounts for 3.4% of global greenhouse gas emissions, with projections indicating a sharp rise if current trends continue [23].

#### 1.1.3. Global Spread and Socioeconomic Implications

Plastic waste is no longer confined to highly urbanized regions or coastal areas. Due to the lightweight and durable nature of plastics, they can be transported by wind, water currents, and even migratory animals. This has led to their widespread distribution in remote areas such as the Arctic, the Himalayas, and uninhabited islands. The discovery of plastic particles in Arctic snow and Antarctic sea ice illustrates the global reach of plastic pollution, demonstrating that no region is immune to its effects [24,25].

The socioeconomic impacts of plastic waste are also significant. Developing countries, particularly in Southeast Asia and Africa, often lack the infrastructure to manage the growing volume of plastic waste, leading to illegal dumping, open burning, and health hazards for local populations. Informal waste pickers, who play a vital role in recycling, are often exposed to hazardous conditions without adequate protection or compensation [26,27]. Furthermore, the tourism and fisheries industries, critical sources of income for many coastal communities, are negatively affected by plastic pollution. Beaches littered with plastic waste deter tourists, while fishing nets entangled with debris result in reduced catches and damaged equipment. The World Bank estimates that the economic cost of marine plastic pollution reaches up to $13 billion annually, taking into account the impact on tourism, fisheries, and clean-up efforts [28].

### 1.2. Agricultural Waste as a Sustainable Resource for Biopolymer Production

The increasing pursuit of environmentally responsible materials has positioned agricultural waste as a valuable and underutilized resource in the development of biopolymers. Each year, vast quantities of residues such as husks, stalks, peels, shells, and fibers are generated from agricultural activities. While these byproducts are rich in biopolymer precursors like cellulose, starch, lignin, and proteins, they are frequently discarded through open burning or left to decompose in landfills. Such practices contribute significantly to air pollution, greenhouse gas emissions, and soil degradation. Utilizing these materials for biopolymer production addresses waste management issues and offers a pathway to reduce the ecological impact of both agriculture and the plastics industry [29,30].

Agricultural residues present an appealing alternative to fossil-based resources due to their wide availability, renewability, and low processing cost. Unlike purpose-grown biomass crops, these materials do not require additional agricultural inputs, such as land, water, or fertilizers, making them a sustainable and economically feasible option. Many of these residues are structurally composed of polysaccharides and proteins that can be extracted through chemical, enzymatic, or mechanical means. Once extracted, these compounds can be engineered into biodegradable films and packaging materials with tailored properties such as enhanced tensile strength, flexibility, and improved resistance to moisture and gases—essential characteristics for food packaging applications [31]. To provide a clearer overview of the actual biopolymer content in various agricultural wastes, Table 1 presents the typical composition of commonly utilized residues for biopolymer extraction, as reported in the literature.

From a sustainability standpoint, converting agricultural waste into functional biopolymers aligns with the principles of the circular economy [38]. Instead of viewing waste as an endpoint, this model promotes its reintegration into the production cycle, thereby optimizing resource use and minimizing environmental burden. Such an approach contributes to broader environmental objectives and supports global initiatives, such as the Sustainable Development Goals, particularly those related to Industry, Innovation, and Infrastructure (Goal 9) and Climate Action (Goal 13) [39,40].

Beyond the environmental benefits, valorizing agricultural waste also presents socio-economic advantages. Farmers and rural communities can benefit from an additional income stream by supplying raw materials previously considered waste. For industry players, producing biopolymers from agricultural residues can reduce dependence on virgin materials, lower production costs, and enhance the sustainability profile of their products. Regulatory bodies and policymakers are increasingly recognizing these benefits, offering financial incentives and support for biowaste-based innovations and cleaner production technologies [40,41].

Recent advancements in biopolymer technology have further enhanced the feasibility of using agricultural waste. Innovations in extraction processes, such as the use of green solvents and ionic liquids, as well as physical and chemical modification techniques, have led to improved material performance. Additionally, integrating nanomaterials and bioactive compounds into biopolymer matrices has enabled the development of multifunctional packaging with antimicrobial, antioxidant, and barrier functionalities [42]. As a result, agricultural waste-derived biopolymers are gaining recognition as promising candidates to replace conventional plastics in food packaging, moving closer to commercial scalability and market acceptance. Several reviews exist on biopolymers and bioplastics; here, we specifically focus on those derived from agricultural waste and their viability for food packaging, an angle that addresses both waste valorization and the use of sustainable materials.

## 2. Types of Biopolymers Derived from Agricultural Waste

The ongoing pursuit of sustainable alternatives to conventional synthetic polymers has significantly intensified interest in biopolymers, especially those derived from renewable and eco-friendly sources. Among these, agricultural waste has emerged as a particularly attractive feedstock due to its abundant availability, low economic value, and high content of biopolymer-forming components. Agricultural residues such as rice husk, wheat straw, sugarcane bagasse, banana peels, corn husks, cassava peels, and spent mushroom substrate contain considerable amounts of cellulose, hemicellulose, lignin, starch, and proteins. These natural macromolecules possess the fundamental structural features required to develop a range of biodegradable materials that can be used in packaging, biomedical, and agricultural applications [43]. This chapter explores the various categories of biopolymers that can be obtained from such residues, focusing on their extraction methods, properties, modification strategies, limitations, and applications.

Agricultural waste, which was once treated merely as a disposal problem, has now gained prominence as a renewable bioresource with significant industrial potential. Globally, millions of tons of crop residues and agro-industrial byproducts are produced annually, much of which remains unutilized or is burned openly, leading to serious environmental issues such as air pollution and greenhouse gas emissions [44]. Transforming these residues into value-added biopolymers not only supports waste valorization and circular economy practices but also mitigates dependence on fossil fuels, reduces plastic pollution, and enhances the sustainability of the packaging industry. The utilization of agricultural waste offers dual benefits: environmental remediation through waste reduction and the development of bio-based materials with desirable functional characteristics.

The term “biopolymer” refers to naturally occurring polymers or those synthesized through biological pathways, which are biodegradable and often biocompatible. These polymers decompose through microbial action into carbon dioxide, water, biomass, and other naturally occurring substances without leaving harmful residues in the environment [45]. Based on their source and chemical nature, biopolymers can be broadly categorized into polysaccharide-based (e.g., cellulose, starch, chitosan), protein-based (e.g., soy protein, whey protein, gelatin), and microbial-derived (e.g., polyhydroxyalkanoates, polylactic acid). The versatility of these polymers in forming films, fibers, and gels makes them ideal for diverse applications [45,46]. However, raw biopolymers derived directly from agricultural residues often require purification, modification, or blending to improve their processability and performance characteristics for commercial use.

The extraction and conversion of these polymers from agricultural waste involve a variety of chemical, physical, and biological techniques. For instance, cellulose can be extracted using alkali treatment, bleaching, and hydrolysis, followed by surface modification techniques such as esterification, etherification, or graft copolymerization to enhance its hydrophobicity and mechanical strength. Similarly, starch extracted from corn, cassava, or potato peels can be plasticized to form flexible films, although its inherent brittleness and sensitivity to moisture require reinforcement through nanofillers or blending with other biopolymers. Protein-based polymers extracted from agro-residues or food processing byproducts, such as soy protein isolate or whey, offer excellent gas barrier properties but are prone to moisture absorption and require crosslinking or blending for improved water resistance and mechanical integrity [47,48].

An emerging area within this field is the biosynthesis of microbial bioplastics such as polyhydroxyalkanoates (PHA) and polylactic acid (PLA), which can also be produced from agricultural waste substrates through fermentation by specialized microorganisms. Although the production costs of these materials remain high, ongoing advancements in microbial engineering, fermentation optimization, and feedstock pre-treatment are gradually making them more commercially viable [49]. These microbial bioplastics represent a bridge between traditional petrochemical-derived plastics and future sustainable packaging solutions, as they exhibit properties comparable to conventional polymers like polyethylene and polypropylene but are fully compostable and non-toxic [49,50].

Despite their promising potential, biopolymers derived from agricultural waste face several challenges that limit their widespread adoption. These include variability in raw material composition depending on geographic and seasonal factors, low yields of extraction, and the need for multi-step processing. Additionally, the mechanical, thermal, and barrier properties of some of these biopolymers may fall short when compared to synthetic counterparts, necessitating further research into functional additives, nanocomposites, and compatibilization strategies [30,51]. Addressing these challenges requires an interdisciplinary approach involving material science, chemical engineering, microbiology, and environmental science to create innovative solutions that enhance the properties and reduce the cost of production.

This section delves into the most relevant categories of biopolymers that can be derived from agricultural waste, offering a detailed exploration of their extraction techniques, physicochemical characteristics, structural modifications, and real-world applications. Section 2.1 discusses cellulose-based biopolymers, emphasizing the extraction and modification of cellulose from various lignocellulosic wastes. Section 2.2 addresses starch-based biopolymers and the critical role of plasticizers and reinforcement techniques in improving film performance. Section 2.3 explores protein-based biopolymers, highlighting their unique gas barrier properties and the strategies used to enhance their water resistance and mechanical strength. Lastly, Section 2.4 focuses on polyhydroxyalkanoates (PHA) and polylactic acid (PLA), covering microbial production using agricultural substrates and potential solutions to overcome production and performance limitations.

As the global community strives for cleaner production technologies and reduced plastic dependency, agricultural waste-derived biopolymers stand out as promising materials for the future. They align closely with the principles of green chemistry, industrial ecology, and sustainable development. With continued innovation and supportive policy frameworks, these materials are well-positioned to play a central role in transitioning toward a bio-based economy.

### 2.1. Cellulose-Based Biopolymers

#### 2.1.1. Introduction to Cellulose and Its Abundance in Agricultural Waste

**Figure 1:**
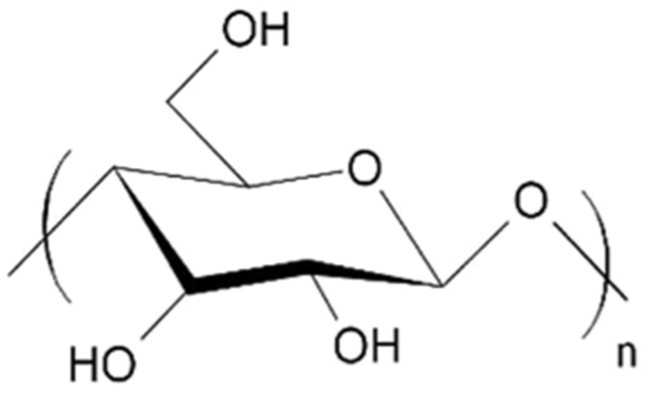
Structure of cellulose.

Cellulose is a naturally occurring linear polysaccharide composed of β-(1→4)-linked D-glucose monomers as shown in Figure 1. As the primary structural component of plant cell walls, it is the most abundant biopolymer on Earth, accounting for nearly half of the dry weight of most plant matter. The cellulose molecule is characterized by a high degree of polymerization, typically ranging from several hundred to over 10,000 glucose units, and forms a rigid, fibrous structure due to extensive intermolecular and intramolecular hydrogen bonding [52,53]. This tightly packed, crystalline arrangement imparts significant tensile strength and thermal stability to plant cell walls, so cellulose has long been valued as a structural material [54].

In recent decades, there has been a growing interest in utilizing cellulose from renewable resources for the development of sustainable materials, particularly in the realm of biodegradable packaging. One of the most promising sources of cellulose lies in the vast quantities of agricultural residues generated globally each year. These lignocellulosic biomass wastes often treated as byproducts or even environmental nuisances include rice husks, sugarcane bagasse, banana peels, wheat straw, corn stalks, oil palm empty fruit bunches, pineapple leaves, coconut shells, and spent mushroom substrate. These materials, though discarded in large volumes, contain a significant proportion of cellulose, often ranging between 30% to 50% by dry weight depending on the source and pre-treatment methods.

The composition of these residues is typically lignocellulosic, comprising three primary components such as cellulose, hemicellulose, and lignin. Cellulose acts as the skeleton of plant tissues, while hemicellulose and lignin contribute to the matrix and rigidity of the biomass [55,56]. With appropriate treatment, cellulose can be efficiently separated from the non-cellulosic fractions, yielding a pure, biodegradable polymer that serves as a viable alternative to conventional petrochemical-based plastics. The utilization of such waste materials not only reduces dependency on non-renewable fossil resources but also adds value to agricultural byproducts that are otherwise incinerated, landfilled, or left to decompose—practices that contribute to greenhouse gas emissions and environmental degradation.

The availability of agricultural waste is particularly significant in countries with large agrarian economies. Globally, agricultural waste amounts to approximately 998 million tons annually. In Malaysia alone, about 1.2 million tons of this waste end up in landfills each year. Across Asia, agro-waste constitutes an estimated 15% of the total waste produced. Specifically in Malaysia, the agricultural waste generation rate in 2009 was around 0.122 kg per person per day, and it is projected to increase to 0.210 kg per person per day by 2025 [57,58]. So, it is estimated that billions of tons of lignocellulosic waste are produced each year, making it a virtually inexhaustible and sustainable source of raw material. Utilizing this biomass for the extraction of cellulose can play a critical role in addressing the challenges of plastic pollution while promoting circular economy principles.

In Malaysia, several companies are actively transforming agricultural waste into sustainable food packaging solutions. Circlepac produces compostable food packaging using agricultural waste such as bamboo and sugarcane bagasse, offering an eco-friendly alternative to single-use plastics. They also provide composting services to help businesses manage organic and packaging waste more sustainably. PXL Marketing is a local supplier of disposable food packaging that includes containers made from sugarcane bagasse, a byproduct of sugar refining. These containers are leakproof, microwave-safe, and suitable for a variety of food types due to the inherent strength of sugarcane fiber, which is rich in cellulose and lignin. Similarly, Zerolife develops meal boxes and biodegradable straws using agricultural residues like bamboo pulp, sugarcane pulp, and reed pulp. Their products are fully home-compostable and break down within 40 days, offering a cost-effective and eco-conscious replacement for plastic and Styrofoam packaging. These initiatives reflect Malaysia’s growing commitment to reducing plastic waste through the upcycling of agro-industrial byproducts.

From a materials science perspective, cellulose offers a wide range of desirable properties that make it suitable for sustainable packaging applications. It is inherently biodegradable, non-toxic, lightweight, and renewable. Moreover, it exhibits good mechanical strength, thermal stability, and film-forming capabilities [59]. When extracted and processed appropriately, cellulose can be fabricated into films, fibers, foams, and composites suitable for packaging dry foods, fresh produce, and consumer goods. Its oxygen barrier properties, when dry, are particularly advantageous for prolonging the shelf life of packaged items [60]. Additionally, the chemical structure of cellulose offers numerous reactive sites, primarily the hydroxyl groups at the C2, C3, and C6 positions of each glucose unit, which can be modified through physical or chemical means to enhance its performance in various applications [61]. These modifications can improve characteristics such as hydrophobicity, flexibility, transparency, thermal resistance, and compatibility with other biopolymers.

The extraction and valorization of cellulose from agricultural waste not only support sustainable material development but also align with several Sustainable Development Goals (SDGs), including Goal 12 (Responsible Consumption and Production) and Goal 13 (Climate Action). Furthermore, converting cellulose into biopolymer packaging materials helps in reducing the environmental footprint associated with synthetic plastics, which are known for their persistence in the environment and their contribution to marine and terrestrial pollution.

In conclusion, cellulose derived from agricultural residues offers a compelling pathway for developing eco-friendly materials, particularly in food packaging sectors. Its abundance, renewability, and modifiability provide a solid foundation for replacing conventional plastics and advancing green technologies. As global attention shifts towards reducing environmental pollution and embracing bio-based solutions, cellulose stands out as a key material in the transition towards sustainable and circular material systems.

#### 2.1.2. Extraction Methods of Cellulose from Agricultural Waste

**Figure 2:**
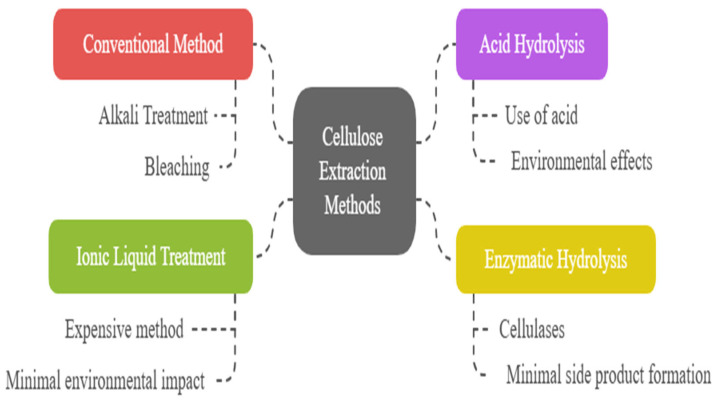
Extraction methods of cellulose.

Agricultural residues such as rice husks, sugarcane bagasse, wheat straw, banana peels, and spent mushroom substrate are composed of three primary biopolymers: cellulose, hemicellulose, and lignin. Among these, cellulose is the target compound for most material applications due to its structural strength, biodegradability, and renewability. However, its isolation requires the removal of surrounding matrix components, primarily lignin and hemicellulose, that shield the cellulose microfibrils within the plant cell wall [62]. The conventional approach for cellulose extraction involves a combination of alkaline treatment followed by bleaching, which effectively deconstructs this complex architecture to release cellulose in its purified form [63]. Figure 2 provides an overview of the different methods employed for extracting cellulose.

(a) Alkaline Treatment

Alkaline treatment is often the first major step used to break down lignocellulosic structures and liberate cellulose fibers. This process primarily targets the removal of hemicellulose and partial delignification by disrupting ester bonds and solubilizing non-cellulosic polysaccharides [64,65]. Typically, sodium hydroxide (NaOH) is employed as the chemical agent. The ground agricultural waste is soaked in an aqueous NaOH solution, commonly in concentrations ranging from 2% to 10%, and heated to temperatures between 60 °C and 90 °C [65,66]. The duration of the treatment typically ranges within a few hours, depending on the type of biomass and its lignin content. Mechanistically, the alkaline environment hydrolyzes the ester linkages that connect hemicellulose to lignin and cellulose. The hydroxide ions (OH^−^) from NaOH attack the carbonyl carbon of ester bonds, leading to saponification reactions and releasing hemicellulose in soluble form. The general reaction mechanism can be represented as the following:

R–COO–R′ + OH^−^ → R–COO^−^ + R′–OH

Here, R–COO–R′ represents the ester linkage in the hemicellulose-lignin complex. As the hemicellulose is removed, the structure becomes more porous, increasing the accessibility of cellulose. In addition, the phenolic groups in lignin are ionized under alkaline conditions, which aids in its partial solubilization. The swelling of biomass fibers also occurs, disrupting hydrogen bonds within the matrix and facilitating the physical release of cellulose fibrils. Upon completion of the treatment, the mixture is filtered to separate the solid residue, now enriched in cellulose, and then washed repeatedly with distilled water until a neutral pH is achieved. This step is crucial to remove residual alkali and solubilized compounds. At this stage, the material still retains a light brown coloration due to the presence of residual lignin and chromophores, necessitating further purification through bleaching [67,68].

(b) Bleaching

To obtain cellulose of high purity and brightness, a bleaching step is performed following the alkaline treatment. Traditional bleaching agents, such as chlorine-based chemicals, pose environmental hazards and can degrade cellulose. Instead, an eco-friendly method involves using bleaching agents such as sodium chlorite (NaClO_2_) and hydrogen peroxide (H_2_O_2_) in combination with glacial acetic acid to achieve effective delignification and removal of chromophores under mildly acidic conditions [69].

In this method, the alkali-treated cellulose is immersed in a solution containing 5–10% hydrogen peroxide. The pH of the solution is adjusted to between 4 and 5 using glacial acetic acid, which also acts as a buffering agent. The reaction mixture is maintained at a temperature of 50 °C to 80 °C for 1 to 2 h, depending on the extent of lignin and pigment removal required. This environment promotes the in situ formation of peracetic acid (CH_3_CO_3_H), a powerful oxidizing agent, through the equilibrium reaction:

H_2_O_2_ + CH_3_COOH ⇌ CH_3_CO_3_H + H_2_O

Peracetic acid oxidizes the lignin molecules by cleaving aromatic rings and breaking the chromophoric structures responsible for coloration. The mechanism involves the generation of reactive oxygen species (ROS), such as hydroxyl radicals (•OH) and perhydroxyl anions (HO_2_^−^), which attack the electron-rich sites of lignin. These oxidative reactions lead to the fragmentation of lignin macromolecules into smaller water-soluble phenolic compounds, which can be easily washed away. Additionally, the process degrades conjugated double bonds and other unsaturated groups responsible for the yellow-brown color of the cellulose, leading to a whiter product [70,71].

An advantage of this method is the controlled pH environment, which prevents excessive cellulose degradation. Unlike high-pH bleaching, which can cause cellulose depolymerization, the mildly acidic conditions help maintain the degree of polymerization (DP) and crystallinity index (CrI) of cellulose. This preservation of molecular integrity is crucial for applications requiring mechanical strength and thermal stability, such as biocomposite films and membranes. After bleaching, the cellulose is thoroughly rinsed with distilled water to remove residual acids, peroxides, and lignin degradation products. The resulting product is a white, fibrous material composed predominantly of cellulose, with minimal traces of non-cellulosic substances. It can be dried at 50–60 °C in a hot air oven or vacuum oven for storage and further use [72,73].

In summary, the conventional two-step process, alkaline treatment followed by acetic acid-assisted peroxide bleaching, offers an efficient, scalable, and environmentally conscious method for isolating cellulose from agricultural residues. Alkaline hydrolysis removes hemicellulose and loosens the lignin structure, while peroxide bleaching under mildly acidic conditions eliminates residual lignin and enhances the whiteness and purity of the cellulose. These methods collectively produce cellulose with improved morphological, chemical, and structural characteristics suitable for further modification or direct use in applications such as biodegradable films, coatings, packaging materials, and nanocomposites.

(c) Acid Hydrolysis

Acid hydrolysis is a widely utilized method for producing cellulose nanocrystals (CNCs) from cellulose that has been previously extracted from agricultural waste. This process effectively removes amorphous regions while preserving the highly ordered crystalline domains of the cellulose, resulting in the formation of CNCs [74,75]. This method typically involves the use of strong mineral acids such as sulfuric acid (H_2_SO_4_), which penetrates the cellulose structure and hydrolyses the β-1,4 glycosidic linkages primarily in the disordered regions. During the process, the acid donates protons to the glycosidic oxygen, leading to the cleavage of the glycosidic bond and the release of shorter, rod-like crystalline fragments. These nanocrystals, typically 100–500 nm in length and 5–20 nm in width, exhibit remarkable crystallinity, mechanical strength, and surface reactivity. The reaction parameters, including acid concentration, temperature (usually between 45–60 °C), hydrolysis duration, and solid-to-liquid ratio, must be carefully optimized to prevent over-degradation and ensure high yield. After hydrolysis, the reaction is quenched with cold water, and the resulting suspension is purified through repeated centrifugation and dialysis to remove excess acid and soluble byproducts [76]. Notably, sulfuric acid introduces sulphate ester groups onto the CNC surfaces, which enhance colloidal stability in aqueous suspensions but may reduce thermal stability. Despite its effectiveness, acid hydrolysis poses environmental and safety challenges due to the corrosive nature of concentrated acids and the generation of acidic waste, necessitating proper neutralization and disposal practices. Nevertheless, CNCs produced via acid hydrolysis are increasingly utilized in biodegradable packaging, nanocomposite reinforcement, barrier films, and pharmaceutical formulations owing to their superior physicochemical properties. Research efforts continue to focus on greener alternatives, such as weaker mineral or organic acids, to achieve more environmentally sustainable nanocellulose extraction while retaining product quality [77,78].

The acid hydrolysis process typically involves the use of a strong mineral acid, most commonly sulfuric acid (H_2_SO_4_) at concentrations ranging from 55% to 65% by weight. When cellulose is suspended in this acidic medium at elevated temperatures (usually 40–60 °C), the hydrogen ions (H^+^) from the acid catalyze the hydrolysis of glycosidic linkages within the amorphous zones of the cellulose chain [79]. These β-1,4 glycosidic bonds are particularly vulnerable in disordered regions due to the increased molecular mobility and exposure to reagents. The hydrolytic cleavage of these glycosidic bonds proceeds via an acid-catalyzed hydrolysis mechanism, where the lone pair of electrons on the glycosidic oxygen accepts a proton from H_3_O^+^, leading to the formation of a highly unstable oxocarbenium ion intermediate. The overall result is the breakdown of the longer cellulose chains into shorter, highly ordered crystalline fragments [80,81]. These fragments, or cellulose nanocrystals, typically have a length ranging from 100 to 500 nm and a width of 5 to 20 nm, depending on the cellulose source and process parameters. The efficiency and quality of CNC production depend heavily on controlling various reaction parameters, such as acid concentration. A higher concentration increases the rate of hydrolysis but also elevates the risk of over-degradation and reduced yields. Temperature plays an important role in acid hydrolysis, where this reaction is typically maintained at 45–60 °C. Higher temperatures accelerate hydrolysis but can promote undesirable side reactions. Reaction durations usually range from 30 min to 2 h. Prolonged exposure leads to cellulose depolymerization and carbonization. Adequate mixing is required to ensure uniform contact between cellulose fibers and acid. Upon the completion of acid hydrolysis, the reaction mixture is quenched using cold distilled water to stop further degradation. The suspension is then subjected to repeated centrifugation and washing cycles to remove excess acid and soluble sugars. Dialysis is often employed to ensure complete removal of sulfate ions and other ionic byproducts. In the case of sulfuric acid hydrolysis, sulfate ester groups may become attached to the surface of CNCs, imparting a negative surface charge and enhancing their dispersion in aqueous media. While this increases colloidal stability, it may also reduce thermal stability due to the presence of acidic sulphate groups [82,83]. Table 2 presents reported yields of nanocrystalline cellulose (NCC) from different agricultural waste sources using the acid hydrolysis method, and the yields are based on the mass of purified cellulose unless otherwise stated.

(d) Enzymatic Hydrolysis

Enzymatic hydrolysis represents an eco-friendly and selective approach for the depolymerization of pre-isolated cellulose from agricultural waste into low molecular weight oligomers, glucose units, or cellulose nanostructures such as cellulose nanofibers (CNFs) and nanocrystals (CNCs), depending on the reaction conditions and enzyme specificity [89]. Unlike acid or alkaline hydrolysis, which involves the use of harsh chemicals and may result in significant degradation or environmental hazards, enzymatic hydrolysis employs cellulase enzymes to cleave the β-1,4 glycosidic linkages in cellulose under mild reaction conditions, typically at neutral pH and moderate temperatures (40–55 °C). This method is desirable for sustainable processing, particularly when derived cellulose is intended for food packaging, and biomedical applications [90].

Cellulases are a group of synergistic enzymes composed mainly of endoglucanases (EGs), exoglucanases (also called cellobiohydrolases, CBHs), and β-glucosidases (BGs). Each of these plays a unique role in the breakdown of cellulose. Endoglucanases randomly cleave internal β-1,4 glycosidic bonds within the amorphous regions of cellulose chains, reducing the degree of polymerization and increasing the number of chain ends [91]. Exoglucanases or cellobiohydrolases act on the terminal ends of the cellulose chains, releasing cellobiose units (a dimer of glucose) from the crystalline regions in a processive manner. β-glucosidases hydrolyze cellobiose and other soluble cello-oligosaccharides into glucose monomers, which can be used in bioethanol or biochemical production [92].

The synergistic interaction between these enzymes is essential for efficient hydrolysis. The reaction proceeds via a retaining or inverting mechanism, depending on the active site configuration of the enzyme. Typically, a general acid/base catalysis mechanism is observed, where two amino acid residues (often carboxylic acids like glutamate or aspartate) are involved, one donating a proton to the glycosidic oxygen and the other acting as a nucleophile or base. Several parameters influence the efficiency of enzymatic hydrolysis, including enzyme concentration. Higher enzyme loading enhances the reaction rate but increases cost, making enzyme recycling or immobilization strategies important in industrial processes. Substrate concentration with optimal cellulose loading prevents inhibition effects due to product accumulation. Enzymes typically function best at pH 4.5–5.5 and 45–55 °C, depending on their source (e.g., Trichoderma reesei, Aspergillus niger, or Thermobifida fusca). Enzymatic hydrolysis is more efficient when agricultural waste is pretreated (e.g., alkali, steam explosion, ionic liquid treatment) to remove non-cellulosic components and enhance enzyme accessibility to the cellulose fraction [93].

Compared to chemical hydrolysis, enzymatic processes are more substrate-specific, resulting in higher selectivity and minimal side-product formation. Moreover, enzymatic methods do not introduce sulfate or other functional groups that might alter the surface chemistry of the cellulose, making them ideal for applications requiring pristine or functionalized nanocellulose with high biocompatibility. The hydrolyzed cellulose obtained via enzymatic methods has several potential uses, particularly in the food, pharmaceutical, biomedical, and cosmetic industries, where chemical residues are unacceptable. Enzymatically produced cellulose nanofibers (CNFs) and nanocrystals (CNCs) exhibit a degree of polymerization, typically ranging from 100 to 800 for CNFs and 100 to 300 for CNCs, which contributes to their excellent biocompatibility and superior dispersion stability in aqueous media [94]. Additionally, the glucose produced as a byproduct can be fermented into bioethanol, contributing to biofuel production [95,96].

(e) Ionic Liquid Treatment

Ionic liquid (IL) treatment has gained considerable attention in recent years as a green and efficient strategy for cellulose dissolution and processing. Unlike conventional extraction techniques that rely on strong acids, alkalis, or high temperatures to break down lignocellulosic biomass, ionic liquids offer a more sustainable alternative due to their low volatility, high thermal stability, recyclability, and non-flammability [97]. These organic salts, which are liquid at or near room temperature, consist of a bulky organic cation (e.g., imidazolium, ammonium, phosphonium) and an inorganic or organic anion (e.g., chloride, acetate, formate). Their unique properties make them highly effective in disrupting the extensive hydrogen bonding network within cellulose, facilitating its dissolution and enabling structural regeneration into desired forms [98].

Cellulose is a semi-crystalline polymer stabilized by a dense network of intra- and intermolecular hydrogen bonds involving the hydroxyl groups of glucose units. These interactions render cellulose insoluble in most conventional solvents. However, certain ILs, especially those containing basic anions like acetate (CH_3_COO^−^), chloride (Cl^−^), and formate (HCOO^−^), can break these hydrogen bonds through strong hydrogen bond acceptor interactions with cellulose hydroxyl groups. The cations, such as 1-ethyl-3-methylimidazolium (EMIM^+^) or 1-butyl-3-methylimidazolium (BMIM^+^), serve to stabilize the resulting dissolved chains by providing electrostatic balance. For example, in 1-ethyl-3-methylimidazolium acetate (EMIMAc), the acetate anion interacts strongly with the –OH groups of cellulose, weakening the interchain hydrogen bonding and allowing the polymer to dissolve. The dissolution is typically conducted at moderate temperatures (60–120 °C), and the cellulose can be regenerated by introducing an anti-solvent such as water, ethanol, or acetone, which precipitates the cellulose as a gel, film, fiber, or powder [99,100].

Advantages of using the IL method are that ILs are typically reusable and exhibit negligible vapor pressure, significantly reducing the emission of volatile organic compounds (VOCs). This method eliminates the need for concentrated sulfuric acid, sodium hydroxide, or organic solvents, minimizing hazardous waste and improving safety. ILs can dissolve a wide variety of cellulose sources, including raw biomass, with minimal or no pre-treatment. The regenerated cellulose often retains high crystallinity and thermal stability, making it suitable for high-performance material applications [101]. IL-treated cellulose can be readily molded into different forms, including membranes, fibers, aerogels, beads, and films, depending on the regeneration pathway. The ability to tailor ionic liquids by modifying the cation-anion pair provides immense flexibility in optimizing dissolution kinetics, cellulose recovery, and product morphology. For example, while acetate-based ILs are excellent for dissolving cellulose with minimal degradation, chloride-based ILs can achieve deeper penetration into crystalline regions. Researchers have also explored dual-function ionic liquids, which not only dissolve cellulose but also introduce functional groups during dissolution, enabling in situ chemical modification [102].

Despite their promising features, ionic liquids are not without drawbacks, such as the synthesis and purification of high-quality ILs remaining expensive, which can be a barrier to industrial scalability. Many ILs are highly viscous, which may limit mass transfer and necessitate dilution with co-solvents or mechanical stirring. Although ILs are often marketed as “green,” some ionic liquids, particularly those with imidazolium or pyridinium cations, can be toxic to aquatic organisms or challenging to degrade in the environment. Ongoing research focuses on biodegradable ILs and deep eutectic solvents (DESs) as more sustainable alternatives. Efficient recycling and reuse of ILs after cellulose regeneration is critical for economic and environmental viability, but this process can be technically demanding [103,104].

#### 2.1.3. Modification Techniques to Enhance Cellulose Properties

Cellulose, a natural polymer derived from plant-based materials, offers a range of desirable properties such as biodegradability, renewability, and mechanical strength, making it an ideal candidate for sustainable food packaging solutions. However, its natural hydrophilicity and limited solubility in most solvents pose challenges in optimizing its performance for food packaging applications. To address these limitations, various modification techniques have been developed to enhance cellulose’s properties, enabling it to meet the demanding requirements of the food packaging industry. These modifications improve key characteristics such as water resistance, mechanical strength, flexibility, and compatibility with other materials, all of which are critical for the development of efficient and sustainable food packaging solutions. By altering the chemical structure of cellulose, these modifications also enhance its barrier properties against moisture, oxygen, and other external factors that can degrade food quality. Chemical modification methods, including esterification, etherification, oxidation, and graft copolymerization, allow for the fine-tuning of cellulose’s properties, making it more suitable for food packaging applications [105,106]. These techniques enable the production of cellulose-based films, coatings, and nanocomposites with improved moisture resistance, mechanical strength, and biodegradability, thereby addressing the growing need for eco-friendly, functional food packaging materials. The following sections will delve into each of these modification techniques, starting with esterification, which is widely used to enhance cellulose’s hydrophobicity and water resistance, crucial for ensuring the longevity and performance of food packaging materials.

(a) Esterification

Esterification is a prominent chemical modification technique that significantly enhances the functional properties of cellulose, particularly by increasing its hydrophobicity and processability, two critical attributes for developing biodegradable films for food packaging. In its native state, cellulose contains abundant hydroxyl (-OH) groups on the C2, C3, and C6 positions of its glucose units. These hydroxyl groups form extensive intra- and intermolecular hydrogen bonds, making cellulose highly hydrophilic and insoluble in most solvents [107,108]. While this natural structure contributes to its mechanical strength and biodegradability, it limits its performance in moisture-rich environments, which is a common condition in food packaging applications. Esterification offers a pathway to overcome these limitations by chemically altering the cellulose backbone, rendering it more suitable for use in packaging films.

**Figure 3:**
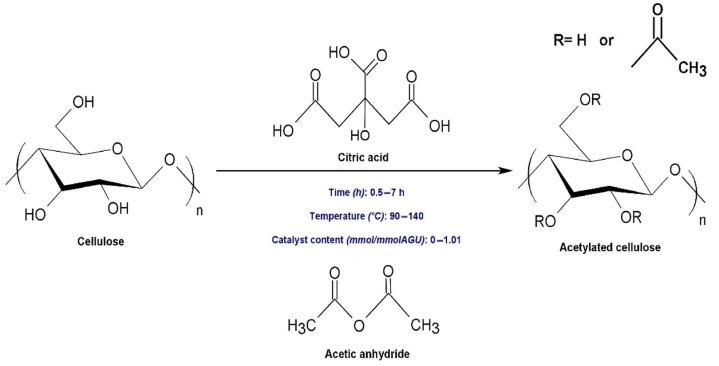
Schematic representation of the esterification process forming cellulose acetate through the reaction of cellulose with acetic anhydride. Reproduced with permission from [111]. Copyright 2016, Elsevier.

The esterification reaction involves substituting the hydroxyl groups of cellulose with ester groups (-COOR) by reacting it with carboxylic acids, acid chlorides, or acid anhydrides as shown in Figure 3. A commonly used reagent in this process is acetic anhydride, and the reaction is typically catalyzed by glacial acetic acid and sulfuric acid. During the reaction, the acetic anhydride donates an acetyl group (CH_3_CO-), which reacts with the nucleophilic oxygen of the hydroxyl group on cellulose, forming an ester linkage and releasing acetic acid as a byproduct. The overall mechanism involves nucleophilic acyl substitution, in which the hydroxyl oxygen attacks the electrophilic carbonyl carbon of the acetic anhydride, displacing the leaving group to form the ester bond [109,110].

This modification results in a product known as cellulose acetate, the most widely studied and commercially available cellulose ester. The degree of substitution (DS) is the average number of hydroxyl groups replaced by ester groups per anhydroglucose unit and plays a crucial role in determining the physical properties of the modified cellulose. The DS of cellulose acetate typically ranges from 0.8 to 3.0. Cellulose diacetate generally has a DS of around 2.0–2.5, while triacetate has a DS close to 3.0, which corresponds to full substitution of the three hydroxyl groups per glucose unit. A DS below 1.5 results in only partial acetylation, which limits hydrophobicity and thermal plasticity [112]. A higher DS leads to a more hydrophobic material with reduced water uptake, enhanced thermal stability, and better film-forming capability. These attributes are particularly important for food packaging, where moisture resistance, mechanical strength, and dimensional stability under varying temperatures are essential [113].

Esterified cellulose, particularly in the form of cellulose acetate films, exhibits improved transparency, flexibility, and surface smoothness, making it ideal for packaging dry foods, baked goods, and fresh produce. These films act as effective barriers to oxygen and water vapor, helping to preserve the freshness and shelf life of food products. Additionally, the surface of cellulose esters can be further treated to incorporate active agents like natural preservatives or antioxidants, expanding their potential beyond passive packaging [114]. In terms of processing, cellulose esters can be easily cast into thin films using conventional solvent-casting or melt-processing techniques. Their thermoplastic nature, unlike native cellulose, allows them to be shaped into various forms such as wraps, pouches, or coating layers. Moreover, their biodegradability ensures that the films break down under appropriate composting conditions, offering an eco-friendly alternative to conventional plastic packaging [115]. Thus, esterification not only addresses the inherent hydrophilicity of cellulose but also unlocks its potential for high-performance, sustainable food packaging applications by improving water resistance, processability, and mechanical integrity.

(b) Etherification

Etherification is another significant chemical modification technique employed to enhance the performance of cellulose, especially in applications such as food packaging where improved solubility, film-forming ability, and environmental stability are crucial. This process involves the substitution of the hydroxyl groups present on the cellulose backbone with ether groups through reactions with alkylating agents such as alkyl halides, epoxides, or chloroacetic acid. Unlike esterification, which introduces ester linkages that can be hydrolyzed under acidic or basic conditions, ether linkages are more chemically stable, offering enhanced durability in various environments [116].

The mechanism of etherification begins with the activation of cellulose hydroxyl groups under alkaline conditions, typically using sodium hydroxide (NaOH). The hydroxyl groups on cellulose are deprotonated to form alkoxide ions (–O^−^), which act as strong nucleophiles. These nucleophilic sites then react with an alkylating agent like monochloroacetic acid, leading to the formation of an ether bond (-O-R) and the elimination of a leaving group (such as chloride ion). A prominent example of this modification is the synthesis of carboxymethyl cellulose (CMC), wherein the etherification of cellulose occurs through reaction with monochloroacetic acid under basic conditions, introducing carboxymethyl (-CH_2_COOH) groups into the structure [117,118].

The introduction of ether groups disrupts the strong hydrogen bonding network within native cellulose, thereby increasing its flexibility and enhancing its solubility in water or other polar solvents. This is particularly beneficial in food packaging applications, where water-dispersible films or coatings are required. CMC, for instance, can be dissolved in water to form clear, uniform films with good mechanical strength, transparency, and barrier properties. These films can act as primary packaging layers for perishable goods, fruits, and vegetables, offering a biodegradable and edible alternative to synthetic polymers [119,120].

Moreover, etherified cellulose films exhibit excellent compatibility with other biodegradable polymers and additives, allowing the development of composite films with tailored properties such as enhanced tensile strength, elongation, and controlled permeability to gases and moisture. In food packaging, such features are vital to maintaining product quality, delaying spoilage, and extending shelf life. Additionally, due to the presence of functional groups like carboxyl groups in CMC, the films can be easily loaded with bioactive compounds, such as antimicrobial agents or natural antioxidants, providing active packaging solutions that interact with food to improve safety and longevity [121,122].

In a study, biodegradable active edible films were developed using okara-derived soluble dietary fiber (SDF), pectin, sodium carboxymethyl cellulose (CMC-Na), and thyme essential oil (TEO). This study focuses on utilizing food processing byproducts to create environmentally friendly packaging materials. The films were fabricated through a solution casting and evaporation technique, and the influence of varying pectin concentrations, with and without TEO, on the structural and functional properties of the films was thoroughly examined. Incorporating TEO generally enhanced the overall performance of the films. Pectin was well integrated into the polymer matrix, contributing to improved film uniformity and interfacial compatibility. Compared to films composed solely of SDF and CMC-Na, those with added pectin demonstrated significantly improved mechanical strength and optical clarity. The highest tensile strength recorded was 21.419 ± 2.22 MPa, while transparency decreased to 88.9% ± 0.42% as pectin content increased. The films also showed better resistance to water and oil permeability. Additionally, they exhibited notable antioxidant activity, achieving a DPPH radical scavenging efficiency of 46.33% ± 0.72%. However, their antibacterial effectiveness against Escherichia coli and Staphylococcus aureus was limited. Overall, these enhanced SDF/pectin/CMC-Na films present promising potential as active, biodegradable edible packaging materials with antioxidant functionality [123].

From a processing standpoint, etherified celluloses can be cast into films using simple aqueous solutions, eliminating the need for toxic organic solvents. This aligns well with environmental sustainability goals and simplifies large-scale production. Furthermore, the resulting films are generally colorless, odorless, and non-toxic, important criteria for materials in direct contact with food [124,125].

**Figure 4:**
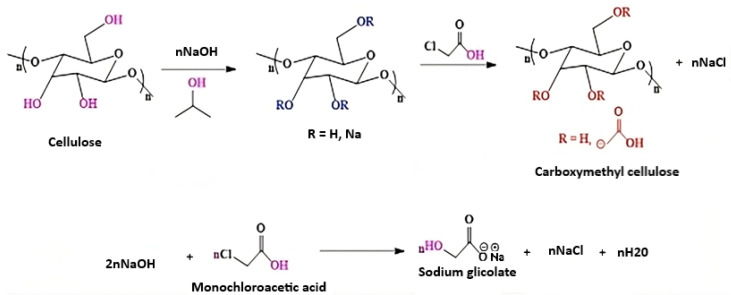
Schematic representation of the etherification process for the synthesis of carboxymethyl cellulose (CMC) from cellulose. Reproduced with permission from [126]. Copyright 2022, John Wiley and Sons.

In summary, etherification improves the water solubility, processability, and functional adaptability of cellulose, making it a valuable approach for developing high-performance, biodegradable films suitable for food packaging applications. These materials offer an environmentally friendly alternative to traditional petroleum-based plastics, with tunable physical and chemical properties. A representative example of this etherification process, in which cellulose is converted to carboxymethyl cellulose (CMC), is illustrated in Figure 4.

(c) Oxidation

Oxidation is a powerful chemical modification technique that enhances the functional reactivity of cellulose by introducing new polar functional groups such as aldehydes, ketones, and carboxylic acids into its molecular structure. This process significantly alters the physicochemical properties of native cellulose, such as its hydrophilicity, surface reactivity, and intermolecular interactions, without compromising its biodegradability. These improvements are particularly valuable for food packaging applications where properties like moisture retention, mechanical flexibility, and compatibility with active agents are critical [127,128].

The mechanism of cellulose oxidation varies depending on the oxidizing agent used, but a widely adopted method is TEMPO-mediated oxidation. In this process, 2,2,6,6-tetramethylpiperidine-1-oxyl (TEMPO) acts as a selective catalyst that converts primary alcohol groups at the C6 position of the glucose unit in cellulose to carboxylic acid groups. In the presence of a co-oxidant system, typically sodium hypochlorite (NaClO) and sodium bromide (NaBr), TEMPO facilitates the one-electron oxidation of hydroxyl groups into aldehydes and further into carboxylates under mild pH and temperature conditions. This high regioselectivity maintains the polymer backbone while increasing the density of negatively charged groups along the cellulose chains [129,130].

Another common method involves periodate oxidation, which cleaves the vicinal diols at C2 and C3 positions to form dialdehyde cellulose. This method introduces aldehyde groups capable of further crosslinking reactions, enabling structural tuning of cellulose materials. Similarly, sodium chlorite and hydrogen peroxide have also been employed to introduce various oxidized functionalities, offering tunable surface energy and chemical reactivity [128,131]. A novel cellulose-based packaging material with exceptional barrier performance and potential application in active packaging has been successfully developed. In this approach, cellulose nanofibrils were selectively oxidized using sodium periodate, introducing reactive aldehyde functionalities. These aldehyde groups contributed to the formation of hemiacetal and hemialdal linkages during film formation, resulting in highly transparent, flexible, and mechanically robust films, even under high humidity conditions. The periodate oxidation process not only altered the chemical structure but also reduced the polarity of the material, significantly enhancing its resistance to water. For example, films treated for 3 and 6 h exhibited water contact angles of 97° and 102°, respectively. Their performance in the water drop test improved markedly, with durations of 138 and 141 min, compared to the untreated sample. Additionally, the water vapor transmission rate (WVTR) decreased to 3.31 and 0.78 g/m^2^/day for 3 and 6 h treatments, respectively. The aldehyde-functionalized surface also enabled the successful immobilization of the enzyme laccase, which acts as an oxygen scavenger to slow down food spoilage. These enzyme-loaded films achieved 80% oxidation of methylene blue dye and maintained catalytic activity over one month and 12 reuse cycles. This work highlights a promising oxidative modification route for cellulose, paving the way for the development of reusable, sustainable alternatives to single-use plastic packaging [132].

From a functional perspective, the oxidized cellulose gains improved hydrophilicity and dispersion ability in aqueous media, making it ideal for forming flexible, transparent films. These films often exhibit enhanced water absorption and swelling properties due to the introduction of ionic carboxyl groups. In food packaging, such films are especially beneficial for wrapping high-moisture products like fresh produce, fish, or bakery items. They help in regulating moisture exchange between the food and the environment, thereby maintaining texture and extending shelf life [133,134].

Additionally, oxidized celluloses act as excellent matrix materials for incorporating antimicrobial or antioxidant compounds. The presence of reactive carboxyl or aldehyde groups allows facile covalent attachment or physical entrapment of bioactive molecules. This makes them ideal candidates for active packaging systems that can inhibit microbial growth or reduce oxidative spoilage in food. For example, aldehyde-rich oxidized cellulose films have been combined with silver nanoparticles or essential oils to produce antimicrobial packaging films with prolonged efficacy [114,135].

Moreover, oxidation enhances the film-forming capabilities of cellulose by increasing its inter-chain hydrogen bonding or enabling crosslinking through secondary reactions. The resulting films exhibit good mechanical strength, improved transparency, and tailored barrier properties. They can also be blended with other biopolymers or plasticizers to modulate flexibility and thermal stability based on packaging needs. Importantly, oxidation processes can be conducted in aqueous media under relatively mild conditions, supporting environmentally benign and scalable fabrication. The oxidized cellulose materials remain biodegradable and non-toxic, complying with the safety standards required for food contact materials [136,137].

In conclusion, oxidation provides a versatile route to functionalize cellulose for use in food packaging films. By increasing chemical reactivity and enhancing physical properties, oxidized cellulose-based materials offer sustainable, high-performance alternatives to synthetic plastics in the packaging industry.

(d) Graft Copolymerization

Graft copolymerization is a chemical modification technique in which side chains of synthetic or natural polymers are covalently bonded onto the backbone of cellulose. This process effectively combines the desirable properties of cellulose, such as biodegradability, mechanical strength, and film-forming ability, with the tailored functionalities of grafted polymers, thereby significantly expanding cellulose’s application potential, especially in the domain of food packaging [138,139].

The mechanism of graft copolymerization involves initiating free radicals on the cellulose backbone, followed by the propagation of monomers to form polymer side chains [140]. Typically, this is achieved using free radical initiators such as ammonium persulfate (APS), ceric ammonium nitrate (CAN), or benzoyl peroxide, which abstract hydrogen atoms from the hydroxyl groups of cellulose, generating active sites for polymerization. Once the radicals are formed, vinyl or acrylic monomers such as methyl methacrylate (MMA), acrylic acid (AA), or acrylonitrile (AN) are introduced, which polymerize and graft onto the activated sites through a chain growth mechanism. This leads to a comb-like structure where cellulose is the backbone and synthetic polymers are the side chains [141].

Alternatively, controlled radical polymerization techniques such as atom transfer radical polymerization (ATRP) and reversible addition-fragmentation chain transfer (RAFT) polymerization have been employed to precisely control graft architecture, grafting density, and molecular weight distribution. These methods offer improved reproducibility and fine-tuning of physical properties, which is critical for packaging applications requiring specific performance characteristics [142,143].

The primary advantage of grafting polymers onto cellulose is the ability to tailor the surface energy, flexibility, water resistance, and barrier properties of the resulting materials. In food packaging, these enhancements are crucial [144]. For instance, grafting hydrophobic polymers such as poly (methyl methacrylate) or polystyrene can significantly reduce the moisture sensitivity of cellulose films, improving their water vapor barrier properties and resistance to deformation in humid environments. This is particularly beneficial for packaging perishable goods, such as dairy products, meats, or ready-to-eat meals, which require materials that can withstand variable storage conditions. Moreover, grafted cellulose materials exhibit improved thermal stability and mechanical strength, allowing them to function effectively as stand-alone packaging films or coatings on paper, cardboard, or biodegradable trays. Their enhanced toughness and flexibility prevent cracking or tearing during handling and transportation, which is often a limitation of pure cellulose films [145,146].

Graft copolymerization also provides a pathway for incorporating functional moieties directly into the cellulose structure. For example, grafting monomers containing functional groups such as carboxylic acids, amines, or quaternary ammonium salts can impart antimicrobial, antioxidant, or oxygen-scavenging properties to the packaging material. This is particularly valuable in the development of active food packaging, where the packaging material plays a role in prolonging shelf life and maintaining food quality [145,147].

Another promising aspect of graft copolymerized cellulose is its compatibility with plasticizers or blending agents, which can be introduced during or after the grafting process to further modify film characteristics [114]. This adaptability ensures that grafted cellulose films can be tailored for specific packaging needs, such as high-barrier outer wraps or breathable inner layers. From an environmental standpoint, graft copolymerization of cellulose offers a sustainable route to high-performance biobased packaging. Unlike petroleum-derived plastics, these materials are inherently biodegradable, and when designed properly, the grafted films can be compostable or recyclable, aligning with circular economy goals in food packaging industries [148,149].

In summary, graft copolymerization is a versatile modification strategy that significantly elevates the performance of cellulose-based materials for food packaging. By tuning the surface chemistry and bulk properties through controlled grafting, cellulose can be transformed into a multifunctional, eco-friendly packaging material that meets modern demands for sustainability, safety, and functionality.

#### 2.1.4. Applications of Cellulose-Based Biopolymers in Food Packaging

Cellulose-based biopolymers have emerged as highly promising alternatives to conventional petroleum-derived plastics in food packaging due to their renewable origin, biodegradability, and tunable functional properties [145]. With growing global awareness of plastic pollution and the pressing need for sustainable materials, the application of cellulose and its chemically or physically modified derivatives in food packaging has gained significant traction [149,150]. The inherent characteristics of cellulose, such as high film-forming ability, mechanical strength, transparency, and chemical reactivity, allow it to be engineered into various packaging formats, including films, coatings, and composites, with properties tailored to specific food preservation needs. Through modification techniques like esterification, etherification, oxidation, and graft copolymerization, the hydrophilicity, thermal stability, and barrier performance of cellulose can be enhanced, making it suitable even for packaging moist, oily, or perishable food products [125,151]. Additionally, advancements in nanotechnology and functionalization have expanded cellulose’s role beyond passive containment to include active and intelligent packaging systems that interact with the food or environment to extend shelf life or indicate spoilage. These developments not only improve food safety and quality but also align with global sustainability goals by reducing environmental impact throughout the packaging life cycle. As a result, cellulose-based biopolymers are increasingly recognized not just as eco-friendly materials but as high-performance packaging solutions capable of meeting the evolving demands of modern food supply chains [152,153].

(a) Biopolymer Films

Cellulose-based biopolymer films, particularly those produced from regenerated cellulose or chemically modified derivatives like cellulose acetate or carboxymethyl cellulose, have garnered significant attention as sustainable alternatives to conventional plastic films used in food packaging. These films exhibit desirable characteristics such as high transparency, tensile strength, biodegradability, and low oxygen permeability, making them well-suited for packaging applications that require both protective and aesthetic functions. Compared to petroleum-based plastic films such as polyethylene (PE) and polypropylene (PP), cellulose films stand out due to their environmentally benign nature and ability to decompose under natural conditions without leaving behind harmful microplastics [154]. While synthetic plastic films often demonstrate superior water resistance and cost efficiency, they pose long-term ecological risks due to their resistance to degradation and contribution to landfill accumulation [155]. In contrast, cellulose films are derived from renewable biomass and can be composted, significantly reducing their environmental footprint. Moreover, their intrinsic ability to form hydrogen bonds contributes to excellent film-forming capabilities and moderate gas barrier properties, particularly against oxygen and grease [156]. However, the hydrophilic nature of unmodified cellulose may limit its water vapor barrier capacity [157]. To overcome this limitation, the addition of plasticizers such as glycerol and sorbitol or the incorporation of cross-linking agents and surface coatings can improve flexibility, processability, and water resistance, thus making cellulose-based films more competitive with synthetic counterparts [158]. Additionally, these films can be tailored for specific applications by incorporating functional additives like antimicrobial agents, antioxidants, or nanofillers, further enhancing their potential in extending food shelf life and ensuring product safety. As consumer demand shifts toward greener packaging, cellulose-based biopolymer films represent a compelling balance between performance and sustainability, making them an attractive choice for eco-conscious food manufacturers [159].

(b) Coatings and Laminates

Cellulose-based coatings and laminates are increasingly employed in food packaging to enhance the barrier, mechanical, and aesthetic properties of paper-based or biodegradable substrates. These coatings typically involve cellulose derivatives such as carboxymethyl cellulose (CMC), hydroxypropyl methylcellulose (HPMC), or cellulose acetate, which can be applied using techniques like dip-coating, spray-coating, or roll-coating [160]. One of the primary advantages of using cellulose-based coatings is their ability to impart moisture resistance and oil repellency to inherently porous and hydrophilic materials like paper, without compromising their biodegradability. In contrast to conventional petroleum-derived coatings such as polyethylene and wax, which hinder compostability and recyclability, cellulose coatings allow the entire packaging structure to remain eco-friendly and suitable for composting [114,161].

Functionally, these coatings act as semi-permeable barriers, limiting the transmission of water vapor, gases, and lipids, thereby improving the preservation and quality of packaged food products. For instance, when used on fruit or vegetable trays, cellulose coatings can reduce dehydration and oxidative spoilage [162]. These coatings can be applied through various techniques, including spraying, dipping, or roll coating, to create multilayered packaging structures with tailored functionalities [163]. Moreover, the coatings can be tailored by incorporating plasticizers to improve flexibility, cross-linkers to enhance structural integrity, or bioactive agents for antimicrobial or antioxidant functionality. In laminated systems, cellulose-based layers can be bonded with other biodegradable films such as starch, gelatin, or polylactic acid (PLA) to form multilayer packaging with complementary properties, for example, combining cellulose’s oxygen barrier performance with PLA’s moisture resistance [164,165].

Additionally, these coatings contribute to packaging aesthetics by providing a smooth, glossy surface finish and can serve as printable layers for labelling and branding. Importantly, because they are derived from renewable agricultural or forestry waste, cellulose coatings align with circular economy goals, offering a low-carbon footprint solution for the food packaging industry [166]. As the demand for compostable and recyclable packaging alternatives grows, cellulose-based coatings and laminates present a highly adaptable and sustainable option for replacing conventional plastic-lined systems.

(c) Nanocomposites

Cellulose-based nanocomposites represent a cutting-edge advancement in sustainable food packaging technology, offering a unique combination of biodegradability, high mechanical strength, and superior barrier properties. These materials are typically fabricated by incorporating cellulose nanocrystals (CNCs) or cellulose nanofibrils (CNFs) into biodegradable polymer matrices such as starch, chitosan, or polyvinyl alcohol (PVA). For instance, CNFs derived from sugarcane bagasse were used as reinforcement in cassava starch-based films at varying concentrations (10% to 50%), significantly improving tensile strength, Young’s modulus, and water vapor barrier properties. Films with 50% CNF content exhibited low water vapor permeability (2.17 g·mm/KPa^−1^·day^−1^·m^2^) and hydrophobic surfaces, making them suitable for food packaging applications such as bakery goods and dry snacks [167]. A notable example of such a nanocomposite is the curcumin-grafted TEMPO-oxidized cellulose nanofiber (CGTOCNF) reinforced chitosan film. In this bio-nanocomposite, CGTOCNF was incorporated at varying concentrations (0–33 wt%) into chitosan matrices. At 10% loading, the nanocomposite film exhibited significantly improved crystallinity (from 21.93% to 87.15%), strong UV-blocking ability, enhanced antibacterial activity, and reduced water solubility. This makes it highly suitable for food packaging applications, particularly for products sensitive to microbial contamination and UV exposure, such as cut fruits, seafood, and fresh poultry [168]. The nanoscale dimensions and high aspect ratio of CNCs and CNFs contribute significantly to the reinforcement of the polymer matrix, resulting in improved tensile strength, stiffness, and resistance to deformation under stress [169,170]. This mechanical enhancement makes cellulose nanocomposites a strong candidate for replacing petroleum-based packaging materials like polyethylene and polypropylene, which dominate the market due to their strength and durability but lack biodegradability.

Regarding barrier performance, the addition of cellulose nanomaterials disrupts the diffusion pathway for gases and moisture, effectively reducing permeability. This is especially beneficial for packaging applications where oxygen and water vapor transmission must be minimized to prolong the shelf life of perishable food items. For instance, the addition of just a small amount of CNCs can significantly lower the oxygen permeability of a biopolymer film, surpassing the performance of some conventional plastics. Unlike synthetic nanoparticles, cellulose nanomaterials used in food packaging are typically derived from renewable agricultural residues such as rice husk, corn stover, and sugarcane bagasse and pose no known toxicity risks, making them safer for direct food contact [171].

Furthermore, the surface chemistry of CNCs and CNFs can be modified to improve compatibility with different polymer matrices or to introduce functional groups that enable further cross-linking, enhancing thermal and moisture resistance. Nanocomposite films can also be engineered to exhibit transparency, flexibility, and printability qualities that are highly valued in consumer packaging [172]. The potential for scalability and customization further enhances the industrial appeal of cellulose-based nanocomposites. Recent innovations also include the development of multilayer films and hybrid nanocomposites, where cellulose nanomaterials are combined with other natural or synthetic fillers to tailor specific performance attributes such as UV shielding, antimicrobial activity, or antioxidant release [173].

In summary, cellulose nanocomposites offer a promising route to high-performance, eco-friendly food packaging that not only addresses the mechanical and barrier limitations of traditional biopolymers but also meets growing consumer and regulatory demands for sustainable materials. As the food packaging industry shifts toward circular and green solutions, cellulose-based nanocomposites stand out as a viable and innovative alternative to conventional plastic packaging.

(d) Active and Intelligent Packaging

Active and intelligent packaging systems have gained significant attention in recent years for their ability to enhance food preservation, improve consumer convenience, and monitor food quality. These packaging systems go beyond the traditional passive role of protecting food from environmental factors by actively interacting with the packaged product or its surrounding environment to preserve freshness, extend shelf life, and reduce food waste [174,175]. Cellulose-based materials, when functionalized with bioactive agents, can be utilized to create innovative active and intelligent packaging solutions, offering a sustainable and biodegradable alternative to conventional synthetic packaging [176].

In active packaging, cellulose-based materials are incorporated with substances such as antimicrobial agents, antioxidants, or enzymes that help preserve the food product. For example, antimicrobial agents like essential oils, chitosan, or silver nanoparticles can be embedded within cellulose-based films or coatings to inhibit the growth of spoilage microorganisms, including bacteria, fungi, and molds [177,178]. This feature is particularly important for perishable products such as meat, dairy, and fresh produce, where microbial contamination can lead to premature spoilage. The inclusion of antioxidants, such as vitamin E or green tea extracts, in cellulose-based packaging can prevent oxidation and prolong the freshness of food items, especially those sensitive to light and oxygen, like oils, nuts, and dairy products. Furthermore, enzymes embedded in the packaging can actively degrade certain compounds, such as ethylene gas, which is responsible for ripening and premature spoilage in fruits and vegetables [179].

Intelligent packaging, on the other hand, is designed to monitor and provide real-time information on the quality or condition of the packaged product. One prominent example is the use of pH-sensitive indicators that change color in response to changes in the acidity or alkalinity of the product. These indicators can be integrated into cellulose-based films or coatings to signal when the food has undergone spoilage or degradation due to microbial activity or environmental factors such as temperature fluctuations [180,181]. Similarly, oxygen or moisture indicators can be embedded in the packaging to detect the presence of gases or humidity levels that may indicate spoilage. Such intelligent features offer consumers a more interactive and informative packaging experience, enabling them to make informed decisions about the freshness and safety of the product [182].

The integration of both active and intelligent features in cellulose-based packaging presents numerous benefits, including reducing food waste by extending product shelf life, ensuring the safety of the food product, and providing valuable information to consumers regarding the quality and safety of the packaged goods [183]. Moreover, these systems offer the added advantage of being biodegradable and sustainable, addressing the increasing concerns about plastic waste and environmental pollution. By combining the inherent biodegradability and mechanical properties of cellulose with functional additives, active and intelligent packaging can play a pivotal role in the development of a more sustainable food packaging industry [184].

In conclusion, the evolution of active and intelligent cellulose-based packaging systems represents a significant advancement in food packaging technology. As demand for sustainability, safety, and consumer convenience continues to grow, cellulose-based active and intelligent packaging solutions are poised to revolutionize the food packaging industry by offering an environmentally friendly, effective, and innovative alternative to conventional petroleum-based materials.

#### 2.1.5. Challenges and Future Perspectives

Despite their potential as sustainable alternatives to petroleum-based plastics, cellulose-based biopolymers face several limitations that must be addressed to enable large-scale adoption in food packaging applications. The intrinsic crystallinity and hydrophilic nature of cellulose restrict its processability and hinder compatibility with hydrophobic polymers, which affects the flexibility, moisture resistance, and mechanical integrity of packaging films [185,186]. Overcoming these issues requires innovative modification techniques to tailor cellulose properties for targeted packaging functionalities. Economic feasibility remains a major challenge. Although cellulose is widely available, current extraction and modification processes often involve high energy input and chemical usage, making them costlier than conventional plastics [187,188]. The scalability of these processes also presents a barrier to commercialization, underscoring the need for energy-efficient and cost-effective production methods [189]. Functional performance is another critical aspect. Cellulose-based materials must offer an optimal balance between biodegradability and key packaging requirements such as tensile strength, flexibility, and gas or moisture barrier performance [190]. Achieving this balance is essential for ensuring product safety and shelf life without compromising environmental advantages. Addressing these trade-offs requires an interdisciplinary approach that integrates materials science, food technology, and environmental engineering to optimize performance without sacrificing sustainability [191]. Future research should prioritize the development of greener extraction methods, including enzymatic treatments and solvent-free or low-impact chemical approaches, to reduce both environmental footprint and production costs [192]. Moreover, incorporating nanomaterials or bioactive additives such as antimicrobial or antioxidant agents can further enhance the functionality and shelf-life of cellulose-based packaging systems [193,194]. The integration of these materials into circular economy models presents a promising pathway toward sustainable packaging. As biodegradable and compostable materials, cellulose-based biopolymers can help reduce plastic waste and align with global sustainability targets by promoting material recovery and end-of-life management strategies [195].

### 2.2. Starch-Based Biopolymers

#### 2.2.1. Sources and Structure of Starch

Starch-based biopolymers have emerged as one of the most promising sustainable alternatives to petroleum-derived plastics in various industries, particularly in the field of food packaging [196]. As global concerns over plastic pollution and its long-lasting environmental impact continue to rise, researchers and manufacturers are increasingly turning towards renewable resources such as starch to develop environmentally friendly materials. Starch, a polysaccharide composed of glucose monomers, is abundant, biodegradable, and readily available from various plant sources [197]. These include corn, potatoes, cassava, and wheat, which are widely cultivated and readily processed into starch [198]. Additionally, agricultural waste materials are gaining attention for the sustainable extraction of starch. Examples of starch-rich agricultural wastes include rice bran, maize husk, potato peels, and banana peels [199]. Utilizing these waste-derived sources not only provides a low-cost raw material but also supports waste valorization and circular economy principles. The potential of starch-based materials, especially films, for use in food packaging, medical products, and other applications is becoming increasingly recognized due to their favorable environmental properties and the growing demand for sustainable alternatives to traditional synthetic polymers [197,200].

Starch is unique because, as a natural biopolymer, it inherently exhibits film-forming abilities, allowing it to be processed into thin films or coatings. These films possess a wide range of properties, such as transparency, flexibility, and biodegradability, which are crucial characteristics for packaging applications. Unlike conventional plastics made from petrochemical derivatives, starch-based biopolymers are capable of breaking down naturally in the environment without leaving harmful residues, thus offering a clear ecological advantage [201]. Furthermore, the use of starch-derived materials promotes the concept of a circular economy, where the production, use, and disposal of materials do not lead to environmental degradation but instead contribute to resource conservation and waste minimization. Despite these advantages, starch-based biopolymers still face several limitations that hinder their direct application in some sectors, particularly in food packaging, where properties such as mechanical strength, water resistance, and stability are critical. This section provides a comprehensive overview of the unique properties of starch-based biopolymers, the challenges they face, and ongoing efforts to enhance their performance for more widespread use [202].

**Figure 5:**
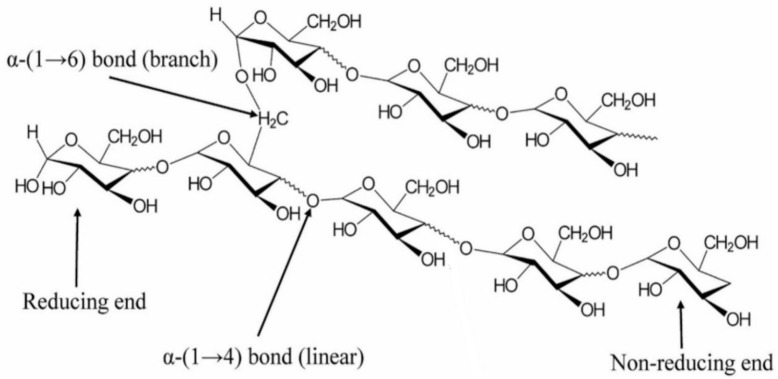
Schematic representation of starch structure showing (1→4)-α glycosidic bonds in the linear regions of amylose and amylopectin and (1→6)-α glycosidic bonds at the branch points in amylopectin. Reproduced with permission from [203]. Copyright 2024, Elsevier.

Starch is a naturally occurring polysaccharide composed of two main components, amylose and amylopectin, both of which are made up of glucose units as shown in Figure 5. Amylose consists primarily of linear chains connected by α-1,4 glycosidic bonds, while amylopectin is highly branched, containing both α-1,4 and α-1,6 linkages at the branching points [203]. The ratio of amylose to amylopectin varies depending on the plant source, but amylopectin is generally more abundant. Structurally, amylose tends to form helical shapes, whereas amylopectin forms a tree-like branched architecture that contributes to the semi-crystalline structure of starch granules [204]. These granules display unique physical characteristics, such as birefringence under polarized light due to their ordered arrangement. When heated in water, starch undergoes gelatinization, a process in which the granules swell, lose their crystallinity, and release amylose, leading to the formation of a viscous gel. Upon cooling, the molecules may reassociate in a process called retrogradation, which affects the texture and stability of starch-based materials [205,206]. These structural and thermal behaviors make starch a widely used material in various industrial and research applications, especially in the development of biodegradable films and sustainable packaging materials.

#### 2.2.2. Film-Forming Ability of Starch-Based Biopolymers

Starch possesses an inherent film-forming ability, making it a widely studied candidate for biodegradable packaging materials. This property originates from the molecular structure of starch, which primarily comprises two polysaccharides: amylose and amylopectin. Amylose, a mostly linear polymer, tends to promote film formation due to its ability to form compact and cohesive networks, whereas amylopectin, with its branched architecture, contributes to flexibility and reduced brittleness. When starch is heated in water, gelatinization occurs, a process that disrupts the granule structure, causing the polymers to leach out and form a viscous paste. Upon drying, this paste transitions into a continuous film with a relatively smooth and transparent surface [202,207].

These films are attractive for applications in food packaging because they are edible, compostable, and derived from renewable sources. Their film-forming capacity can be modulated by adjusting the ratio of amylose to amylopectin, the gelatinization temperature, drying conditions, and the inclusion of plasticizers or other additives. High-amylose starches typically yield stronger, more brittle films, while higher amylopectin content results in softer, more flexible films. Starch films are known to exhibit excellent oxygen barrier properties in dry conditions, which makes them suitable for packaging low-moisture food items [208].

However, pure starch films are highly sensitive to environmental humidity due to the hydrophilic nature of starch. Water molecules can penetrate the film matrix and disrupt intermolecular hydrogen bonds, thereby affecting mechanical properties and dimensional stability. For this reason, although starch films can be formed easily and sustainably, their application is often limited without modification or blending with other materials [209,210].

#### 2.2.3. Challenges Associated with Starch-Based Biopolymers

Despite the many attractive features of starch-based materials, their practical applications, especially in food packaging, are hindered by several critical challenges:

(a) Poor Water Resistance

Starch’s strong affinity for water is one of its main limitations. As a hydrophilic polymer, it absorbs moisture from the atmosphere and readily dissolves in water under certain conditions. This susceptibility significantly limits its use in humid environments or with moisture-rich products. The films tend to lose structural integrity and degrade prematurely when exposed to water, thereby compromising their function as packaging materials [211,212].

(b) Low Mechanical Strength

Starch films are inherently brittle and fragile, particularly in the absence of additives. Their tensile strength and elongation at break are considerably lower compared to synthetic polymers like polyethylene or polypropylene. This restricts their use in applications that require robustness, flexibility, or the ability to withstand mechanical stress during handling, transportation, or storage [213,214].

(c) Retrogradation

Retrogradation is a time-dependent phenomenon in which gelatinized starch molecules, particularly amylose, reassociate and recrystallize upon cooling and storage. This results in the stiffening and embrittlement of starch films over time. Retrogradation not only affects the mechanical and barrier properties of the films but also reduces their shelf stability and processability [215].

(d) Limited Thermal Stability

Starch-based films have relatively low thermal resistance, limiting their application in high-temperature processes such as heat sealing or hot-filling in the food industry. This also makes storage and transportation under fluctuating temperatures a challenge [216,217].

(e) Processability and Industrial Scalability

The processing of starch requires precise control of parameters such as temperature, humidity, and plasticizer content. In industrial applications, ensuring reproducibility and scalability while maintaining film quality can be difficult. Furthermore, the high gelatinization temperature and viscosity of starch can hinder its blending with other polymers or its processing via standard extrusion or injection molding techniques [218].

#### 2.2.4. Strategies to Overcome Challenges

To make starch-based biopolymers more viable for commercial applications, especially in packaging, numerous strategies have been investigated. These approaches aim to enhance mechanical performance, water resistance, thermal stability, and film durability.

(a) Use of Plasticizers

Plasticizers such as glycerol, sorbitol, and polyethylene glycol are commonly incorporated into starch formulations to increase film flexibility. These low-molecular-weight compounds insert themselves between starch chains, reducing hydrogen bonding and increasing molecular mobility. This results in more pliable films with reduced brittleness. However, excessive plasticizer content can lead to increased water uptake and reduced tensile strength, so optimization is critical [219]. For example, a representative study demonstrating the role of plasticizers in enhancing starch-based biodegradable films involved the incorporation of xanthan gum (XG) with starch and the application of various plasticizers, including glycerol, sorbitol, and citric acid. The findings showed that these plasticizers improved the flexibility and structural integrity of the films by disrupting the native intermolecular bonds between XG and starch, leading to a reorganization of hydrogen bonding within the matrix. Glycerol was found to be particularly effective in increasing the film’s elasticity, while sorbitol helped maintain mechanical strength. Surface texture analysis indicated that sorbitol contributed to the formation of smoother films with fewer surface irregularities. Furthermore, a concentration of 1.5% plasticizer was identified as optimal, offering a suitable balance between stickiness, mechanical performance, and surface properties. This study serves as a useful example of how plasticizers can be strategically selected and optimized to improve the functionality of starch-based film materials [220].

(b) Cross-Linking Techniques

Chemical and enzymatic cross-linking strategies are employed to improve the water resistance and mechanical integrity of starch films. Cross-linkers like glutaraldehyde, citric acid, and epichlorohydrin form covalent or ionic bonds between starch molecules, reinforcing the polymer network and enhancing durability. This reduces retrogradation and water solubility while improving the thermal and mechanical properties of the films [221,222]. An illustrative study showcasing the use of crosslinking to enhance starch-based films for food packaging involved the development of composite films made from chitosan, modified cassava starch, and curcumin. These films were fabricated using the solvent casting method, with citric acid (CA) introduced as a natural crosslinking agent at varying concentrations (0–10% by weight based on the dry content of chitosan and starch). The incorporation of CA led to notable improvements in the films’ structural integrity and functional performance. FTIR analysis confirmed the formation of crosslinks between chitosan and starch, indicating successful chemical interaction. The crosslinked films exhibited enhanced water resistance and improved mechanical strength compared to non-crosslinked samples. Notably, a CA concentration of 7.5% yielded the most favorable balance, increasing tensile strength from 8 ± 1 MPa to 12 ± 1 MPa while reducing water vapor permeability, swelling degree, and solubility. However, the antioxidant activity of the films slightly declined with higher CA content. This study effectively demonstrates how citric acid-mediated crosslinking can be utilized to strengthen the mechanical and barrier properties of starch-based films, supporting their application in sustainable food packaging [223].

(c) Blending with Other Biopolymers

Blending starch with complementary biopolymers can mitigate its limitations. For instance, chitosan-starch blends show improved antimicrobial activity and enhanced film strength [224]. Gelatin-starch films demonstrate improved flexibility and moisture barrier properties. Polyvinyl alcohol (PVA)-starch composites offer superior film-forming characteristics and enhanced tensile strength, making them suitable for water-soluble packaging applications [225]. By forming synergistic interactions with these polymers, starch-based composites can achieve a balance between biodegradability, mechanical performance, and functionality. For example, a study demonstrating the blending of starch with other biopolymers to enhance its properties for food packaging involved the development of films using corn starch and chitosan. The films were prepared by combining starch and chitosan solutions in equal volumes through a casting process. The objective was to evaluate how varying chitosan concentrations (0 to 81 wt% relative to starch) influenced the films’ physicochemical, mechanical, and barrier properties, as well as their morphology. Interactions between starch and chitosan molecules were evidenced by FTIR spectral shifts and a decrease in crystallinity, indicating hydrogen bonding and structural modification. The addition of chitosan led to improved tensile strength and elongation at break, with optimal flexibility observed at a concentration of 41 wt%. At higher chitosan levels, however, flexibility slightly decreased. The films also showed increased solubility and moisture content, while Young’s modulus and water vapor permeability were reduced. These findings highlight the potential of starch-chitosan blends in producing biodegradable films with enhanced performance suitable for use in food and pharmaceutical packaging applications [226].

(d) Incorporation of Nanofillers

Adding nanoscale fillers such as nanocellulose, montmorillonite (MMT), or nano-silica into starch matrices significantly enhances barrier, mechanical, and thermal properties. Nanocellulose, in particular, has emerged as a highly effective reinforcement agent due to its high aspect ratio and strong hydrogen bonding with starch chains. These nanofillers form a tortuous path for water vapor and gases, thereby improving barrier performance. Additionally, their reinforcing effect increases tensile strength and thermal resistance without compromising biodegradability [227,228]. An example of enhancing starch-based films for food packaging through nanofiller incorporation involves the addition of hydrophobic silica nanoparticles to corn starch formulations. This study focused on evaluating how varying amounts of amorphous hydrophobic nano-silica (Aerosil^®^ R972, Evonik, Essen, Germany) affected the mechanical behavior, surface energy, surface texture, and overall morphology of the films. The inclusion of silica was found to influence the physical and mechanical performance of the material. Films with lower glycerol and silica concentrations exhibited reduced surface free energy, while those with higher levels showed an increase. Mechanical testing revealed that increased silica content led to a reduction in tensile strength but enhanced flexibility. Additionally, surface roughness was observed to rise with higher silica loading. Scanning electron microscopy (SEM) and energy-dispersive spectroscopy (EDS) confirmed the uniform distribution of the silica particles within the film matrix. This study demonstrates that the incorporation of hydrophobic nano-silica can effectively modify the structural and surface characteristics of starch films, supporting their use as biodegradable alternatives in food packaging applications [229].

(e) Enzymatic and Chemical Modification of Starch

Functionalization of starch through acetylation, oxidation, or grafting with other chemical groups (e.g., ester or ether linkages) can alter its hydrophilicity, crystallinity, and thermal properties. For example, acetylated starch exhibits enhanced water resistance and processability. Such modifications allow the production of tailored starch derivatives that meet specific performance requirements in food and non-food applications [230,231]. A notable example of starch modification to enhance its properties for food packaging involved the development of a hydrophobic starch-based film incorporating alkyl ketene dimers (AKDs) and chitosan. This film was specifically designed for extending the shelf life of mangoes. Through various characterization methods, including FTIR, XRD, XPS, and SEM, the successful integration of AKDs into the starch matrix was confirmed, with esterification leading to the formation of β-ketoester bonds. The modification significantly improved the film’s hydrophobic nature and mechanical strength, attributed to both chemical crosslinking and enhanced hydrogen bonding. When tested over eight days at room temperature, the modified starch film extended mango freshness by an additional four days compared to unmodified films. Given the biodegradable and environmentally friendly nature of the film’s components, this study highlights the potential of chemical modification, such as AKD incorporation, in developing sustainable packaging materials with improved protective functions [232].

### 2.3. Protein-Based Biopolymers

Protein-based biopolymers have attracted increasing interest as sustainable alternatives to synthetic plastics due to their film-forming capabilities, biodegradability, and excellent barrier properties. These materials are typically derived from natural sources such as soy protein, whey protein, casein, gelatin, and wheat gluten, each contributing unique characteristics to the resulting biopolymer films. In addition to traditional protein sources, various agricultural and food processing wastes are also being explored for protein extraction. Examples include soy and sunflower oilseed cakes (residues from oil extraction), wheat bran, rice bran, corn gluten meal, and waste from fish or poultry processing. These byproducts are rich in proteins and present a cost-effective, sustainable alternative for developing protein-based films [233]. The functional properties of protein-based films arise from the complex structure of proteins, which contain both hydrophilic and hydrophobic amino acid residues, allowing for tunable interactions within the polymer matrix [234,235].

#### 2.3.1. Sources and Characteristics

(a) Soy Protein

**Figure 6:**
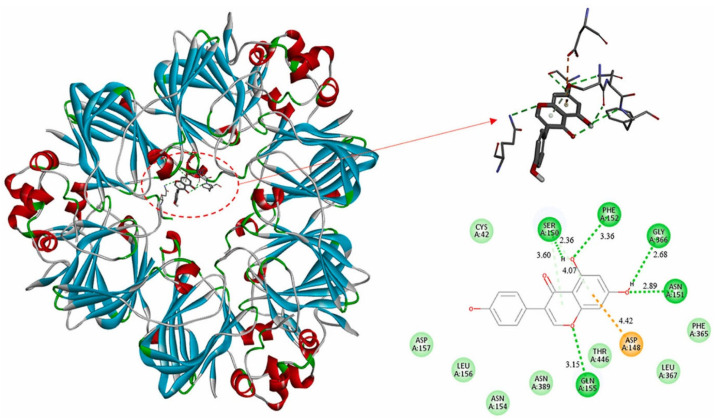
3D view of the optimal binding between glycinin and genistein under normal conditions. The 2D diagram (bottom right) shows interactions with amino acids, including hydrogen bonds (dark green), hydrogen–π interactions (light green), π–anion bonds (orange), and other non-covalent interactions. Reproduced with permission from [239]. Copyright 2023, Elsevier.

Soy protein is one of the most widely studied plant-based proteins in biodegradable film production. Composed primarily of globular proteins such as glycinin and β-conglycinin, soy protein has a high protein content (around 90%) when isolated [236]. Soy protein isolate (SPI) exhibits good film-forming ability and forms transparent, flexible films with notable oxygen barrier properties. However, its sensitivity to moisture and brittleness necessitate modification for practical packaging applications [237,238]. The structure of soy protein is depicted in Figure 6.

Recent developments in protein-based biopolymers have explored innovative methods to enhance the performance of soy protein materials for food packaging. A notable approach involves incorporating natural antioxidants such as grape seed and green tea extracts into soy protein isolate (SPI) formulations. Using 3D printing techniques, researchers have fabricated edible films containing varying concentrations of these extracts (0–5% *w*/*w*), resulting in improved mechanical strength and significantly lower water vapor permeability up to a 61% reduction with grape seed extract and 56% with green tea extract. This method not only boosts the barrier properties of the films but also enables greater design flexibility, allowing the production of customizable packaging materials suitable for real-time application in food preservation [240].

(b) Whey Protein

**Figure 7:**
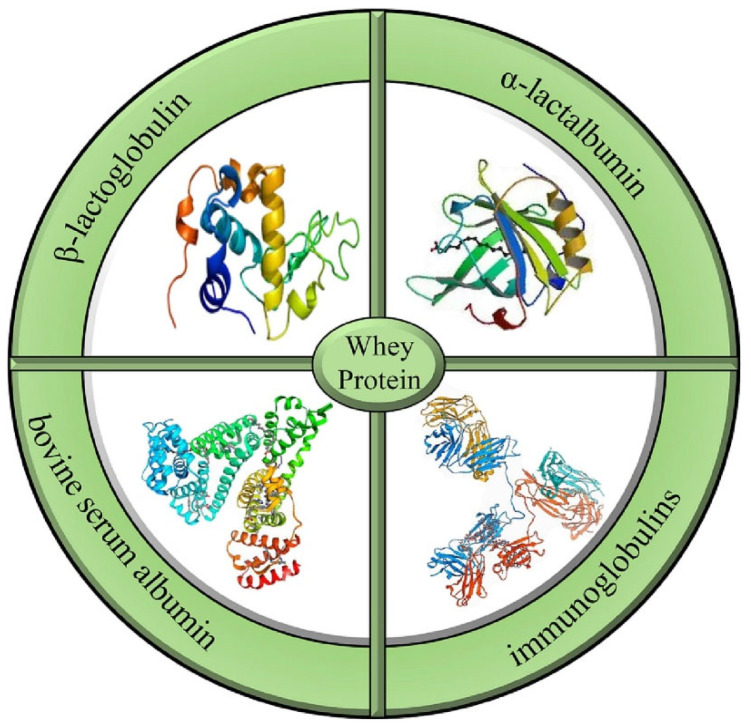
The structure of whey protein. Reproduced with permission from [244]. Copyright 2023, Elsevier.

Whey protein, a byproduct of cheese production, contains β-lactoglobulin and α-lactalbumin as its primary components. It offers superior film transparency, gloss, and excellent oxygen barrier performance [241]. Whey protein films are often used for food coatings and packaging where clarity and low oxygen permeability are essential. However, their poor water resistance and mechanical strength limit widespread adoption unless blended with other materials or plasticizers [242,243]. The structure of whey protein is shown in Figure 7.

Whey protein concentrate (WPC) films have shown promise as biodegradable packaging materials; however, their weak mechanical properties limit their industrial application. Recent studies have investigated the enhancement of these films by incorporating silver nanoparticles (AgNPs) synthesized biologically using *Aspergillus niger*. When added at concentrations of 0.25 to 1.25 mM, the AgNPs significantly improved the strength and water resistance of WPC films, with tensile strength and barrier properties increasing by 84% and 67%, respectively. Moreover, the films exhibited strong antimicrobial effects against several foodborne microorganisms, including *Staphylococcus aureus*, *E. coli* O157:H7, *Salmonella enteritidis*, and *Listeria monocytogenes*, as well as certain fungi and yeasts, showing inhibition zones up to 19.7 mm. Although flexibility was reduced at higher nanoparticle levels, this modification presents a viable approach to developing WPC-based films with enhanced durability and antimicrobial functionality for active food packaging [245].

(c) Casein

**Figure 8:**
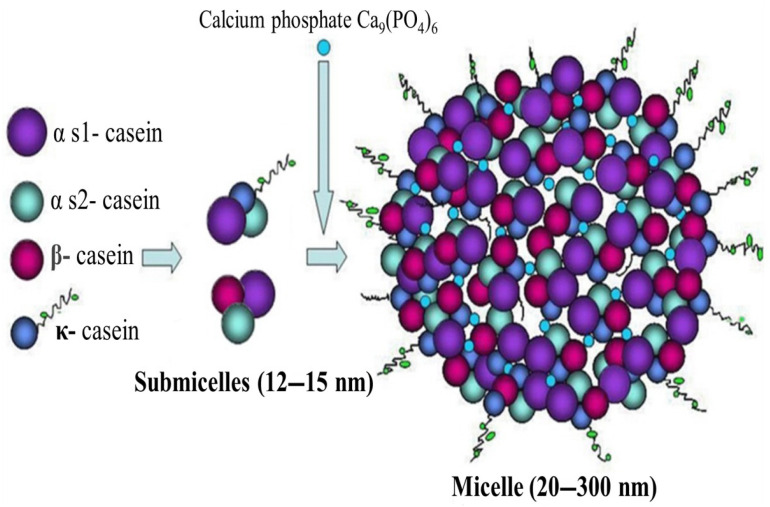
The schematic representation of the casein micelle. Reproduced with permission from [246]. Copyright 2017, John Wiley and Sons.

Derived from milk, casein is a phosphoprotein that forms strong and flexible films. It is mainly composed of four distinct proteins: αs1-casein, αs2-casein, β-casein, and κ-casein as shown in Figure 8.

Casein films are edible and tasteless and exhibit good gas barrier properties, making them ideal for food coatings and pharmaceutical packaging applications. However, similar to other protein films, they suffer from poor resistance to water and humidity, requiring reinforcement or surface treatment [247,248]. Casein-based films have gained considerable attention in the field of sustainable food packaging due to their natural origin and excellent film-forming ability. When combined with polysaccharides like guar gum, their functional properties can be further enhanced. A recent study demonstrated that incorporating gallic acid into a sodium caseinate-guar gum film matrix significantly improved the material’s performance. At lower concentrations, gallic acid reduced the film’s water vapor permeability by 21%, which enhances its barrier function. Simultaneously, a slight increase in water solubility from 58% to 63% was observed, potentially accelerating the film’s biodegradation. In addition, the films showed high antioxidant activity, with about 80% DPPH radical inhibition, indicating their suitability for active food packaging applications where oxidative protection is required [249].

(d) Gelatin

**Figure 9:**
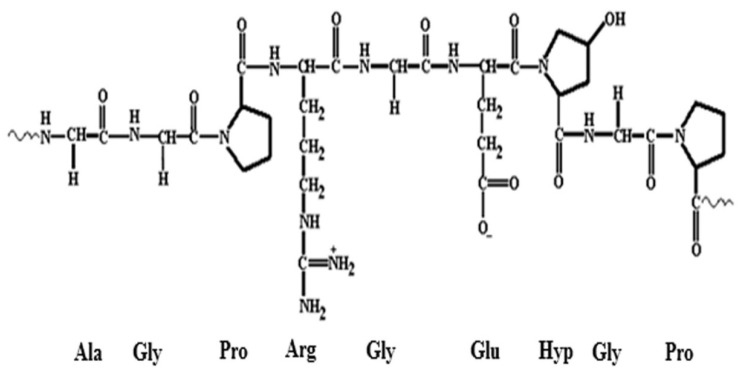
The chemical structure of gelatin. Reproduced with permission from [250]. Copyright 2017, Elsevier.

Gelatin is produced by the partial hydrolysis of collagen from animal connective tissues, with its chemical structure illustrated in Figure 9.

Gelatin is a thermoreversible gelling agent that forms transparent, smooth, and flexible films. Gelatin films are widely used in edible packaging due to their high clarity, good aroma barrier properties, and ease of processing. Yet, they are also highly hygroscopic and can become sticky or brittle depending on the surrounding humidity [251,252]. Recent advancements have explored the functional enhancement of gelatin films through the incorporation of natural extracts and nanomaterials. In one such study, grapefruit seed extract (GSE) and titanium dioxide (TiO_2_) nanoparticles were added to gelatin to improve its performance. The optimized formulation containing 0.5 wt% TiO_2_ exhibited the best mechanical strength and water resistance, while also significantly reducing water vapor permeability. GSE contributed to the antioxidant and antibacterial properties of the film, particularly against *E. coli* and *L. monocytogenes*, while TiO_2_ provided effective UV-blocking capabilities. These multifunctional properties make gelatin-based films reinforced with GSE and TiO_2_ promising candidates for use in active packaging, where both microbial protection and UV shielding are essential for extending food shelf life [253].

(e) Gluten

**Figure 10:**
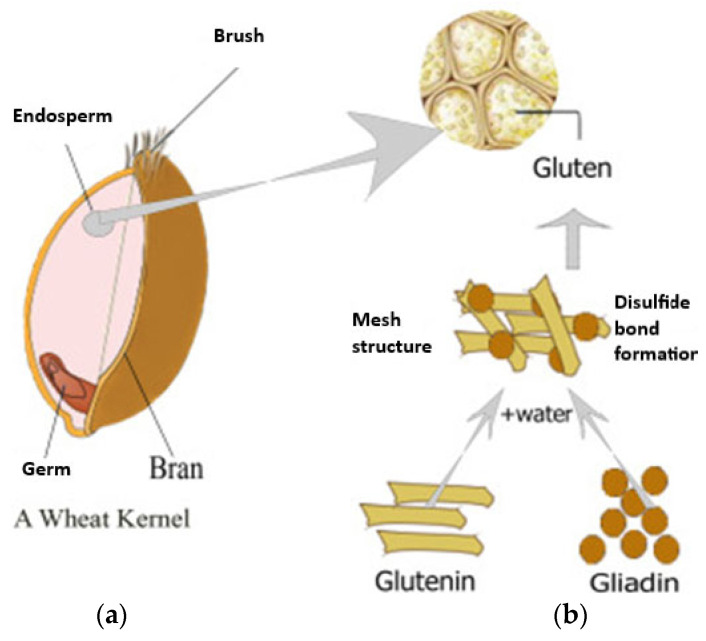
Schematic representation of (**a**) gluten protein structures and (**b**) gluten in dough. Reproduced with permission from [256]. Copyright 2023, Elsevier.

Wheat gluten is a protein complex composed mainly of gliadin and glutenin. It exhibits excellent film-forming capabilities and has been explored for agricultural and food packaging applications [254]. Gluten films show reasonable mechanical strength and elasticity but are limited by their water solubility and sensitivity to environmental moisture [255]. The gluten structure is shown in Figure 10.

A recent study enhanced wheat gluten films by incorporating chlorophyll and polypyrrole, aiming to improve their functional properties. The resulting composite films exhibited reduced solubility and improved tensile strength, making them more durable for packaging applications. Opacity and antioxidant activity increased with both additives, while polypyrrole specifically contributed to strong antibacterial effects against Escherichia coli. Structural analysis confirmed new interactions among the components, and surface imaging showed uniform dispersion of polypyrrole particles across the film. Interestingly, the film’s conductivity and color also changed over time, suggesting potential as an intelligent packaging material capable of indicating food freshness. These enhancements highlight the suitability of modified wheat gluten films for smart and active packaging systems [257].

#### 2.3.2. Advantages of Protein-Based Biopolymers

Protein-based films offer several compelling advantages, particularly for use in food packaging. Due to their dense polymeric structure, protein-based films can effectively block oxygen transmission, thereby protecting food products from oxidative degradation and prolonging shelf life. Many protein-based films are not only biodegradable but also edible, making them suitable for direct-contact food applications without contributing to environmental waste. These films often exhibit high visual appeal due to their smooth texture and optical clarity, which is advantageous for food packaging that requires product visibility. Proteins can be sourced from agro-industrial byproducts, promoting resource efficiency and circular economy practices [243,258].

#### 2.3.3. Limitations of Protein-Based Biopolymers

Despite their desirable properties, protein-based biopolymers face several challenges that must be addressed before large-scale commercialization. Proteins contain numerous polar functional groups capable of forming hydrogen bonds with water molecules. As a result, protein-based films are highly hydrophilic, making them unsuitable for moist environments unless modified [259]. Native protein films often display low tensile strength and limited elongation at break. This restricts their use in applications requiring structural robustness or flexibility. Protein films typically lack sufficient thermal resistance, which limits their application in heat-intensive processes such as sterilization or hot filling. Films tend to shrink or crack upon drying, especially when improperly plasticized or when subjected to rapid dehydration [260,261].

#### 2.3.4. Strategies for Property Enhancement

Various physical, chemical, and biological modification techniques have been employed to improve their functional properties, particularly mechanical strength, water resistance, and film stability to overcome the inherent drawbacks of protein-based biopolymers. One widely adopted approach is cross-linking, which involves forming covalent or non-covalent bonds between protein chains to enhance the cohesive strength of the matrix. Chemical cross-linkers such as aldehydes (e.g., glutaraldehyde), tannic acid, and citric acid are effective in forming stable intermolecular networks, while enzymatic methods, including the use of transglutaminase and laccase, offer eco-friendly alternatives without introducing toxic residues [262,263]. Another strategy is polymer blending, where proteins are combined with polysaccharides such as starch, chitosan, pectin, or cellulose derivatives to create a composite structure with balanced mechanical and barrier properties. These combinations often result in improved film flexibility, reduced water solubility, and enhanced durability due to synergistic interactions between the two biopolymer matrices [264,265]. The addition of plasticizers such as glycerol, sorbitol, or polyethylene glycol is also common; these substances reduce intermolecular forces, increase molecular mobility, and enhance flexibility, though excessive use may reduce tensile strength and increase permeability [265,266]. To further reinforce the structural integrity of protein-based films, nanofillers such as nanocellulose, montmorillonite clay, and graphene oxide are incorporated into the matrix to create nanocomposites with superior barrier properties, thermal stability, and mechanical performance by forming tortuous pathways that slow down the diffusion of gases and water vapor [267]. Moreover, surface treatments and lamination techniques, such as coating the protein film with lipids, waxes, or biodegradable polymers like polylactic acid, can impart hydrophobicity and moisture resistance, making the films more suitable for practical food packaging applications [268,269]. These enhancements collectively contribute to the growing viability of protein-based biopolymers as functional, sustainable packaging materials capable of replacing petroleum-based plastics under appropriate conditions.

### 2.4. Agricultural Residue-Derived Bioplastics: PHA and PLA

#### 2.4.1. Sources of PHA and PLA

Bioplastics such as polyhydroxyalkanoates (PHA) and polylactic acid (PLA) have emerged as leading candidates in the realm of sustainable packaging, particularly in food packaging applications [270,271]. These polymers are primarily synthesized through microbial fermentation processes, utilizing renewable carbon sources derived from agricultural residues such as corn stover, sugarcane bagasse, rice husks, fruit peels, and other lignocellulosic wastes [272,273]. The valorization of such residues not only addresses waste management issues but also enhances the environmental profile of bioplastic production by reducing reliance on food crops and minimizing the carbon footprint associated with traditional feedstocks. PHA is a family of polyesters naturally accumulated by various bacterial strains under nutrient-limited conditions, whereas PLA is a thermoplastic aliphatic polyester obtained through the polymerization of lactic acid, a product of microbial fermentation of carbohydrates. Both materials are biodegradable and compostable under industrial composting conditions, offering a promising end-of-life solution that solves the issue with conventional petroleum-based plastics [49,274].

#### 2.4.2. Advantages of PHA and PLA in Food Packaging

The advantageous properties of PHA and PLA for food packaging applications are closely linked to their intrinsic chemical structures and molecular architectures. PHA, being a family of microbially synthesized polyesters, consists of hydroxyalkanoate monomers connected by ester linkages, which contribute to its biodegradability and hydrolytic sensitivity. The diversity of monomer units—ranging from short-chain-length to medium-chain-length PHAs, allows for tunable mechanical properties, such as flexibility, toughness, and thermal behavior, depending on the monomer composition. For example, poly(3-hydroxybutyrate) (PHB), a common type of PHA, has a highly crystalline structure that imparts excellent barrier properties and stiffness, making it suitable for rigid food packaging [275,276]. PLA, in contrast, is a linear aliphatic polyester derived from lactic acid monomers, where the stereochemistry (L- or D-lactic acid) plays a crucial role in defining its crystallinity, melting temperature, and degradation rate. The regular and stereoregular arrangement of monomers in PLA results in high transparency and tensile strength, qualities desirable in packaging films and containers. Furthermore, the presence of ester bonds in both polymers renders them susceptible to microbial and enzymatic degradation, ensuring complete compostability under suitable conditions. These structural features not only enable their safe return to the environment post-use but also support functional requirements such as oil resistance, moderate water vapor permeability, and compatibility with food-contact standards [277,278]. Overall, the molecular design of PHA and PLA underpins their performance and sustainability, making them strong contenders in replacing conventional plastics.

#### 2.4.3. Limitations of PHA and PLA

Despite these benefits, both PHA and PLA face critical limitations that hinder their commercial scalability and broader industrial adoption. The primary challenge is the high production cost, which is largely attributed to the expensive fermentation processes, limited availability of cost-effective microbial strains, and the need for nutrient-rich substrates [279]. For PLA, while mechanical properties such as tensile strength and clarity are comparable to conventional plastics, it often suffers from brittleness and low elongation at break, restricting its use in flexible packaging formats [280]. PHA, on the other hand, while exhibiting superior biodegradability, has a narrow thermal processing window and is prone to thermal degradation, which complicates its manufacturing and limits its application under high-temperature conditions. Moreover, the purification of PHA from microbial biomass adds additional steps and costs to the production process [281,282]. These factors collectively limit the economic competitiveness of PHA and PLA when compared to low-cost petroleum-based alternatives.

#### 2.4.4. Future Directions

To overcome the above limitations and enhance the feasibility of PHA and PLA in food packaging markets, researchers are actively exploring several future directions. One promising avenue is the genetic engineering of microbial strains, which can improve the yield, efficiency, and tolerance of microorganisms involved in PHA and PLA biosynthesis. Advanced metabolic engineering and synthetic biology approaches are being used to design bacteria and yeasts capable of producing higher amounts of polymers from inexpensive substrates [280,283]. Additionally, the utilization of lignocellulosic agricultural residues as feedstock can significantly reduce substrate costs and alleviate competition with food sources. Pre-treatment technologies, such as enzymatic hydrolysis and ionic liquid-assisted saccharification, are being developed to extract fermentable sugars from biomass more efficiently [284]. Furthermore, blending PHA and PLA with plasticizers, natural fibers, or other biodegradable polymers is being investigated to improve their mechanical flexibility and processing characteristics. Efforts are also underway to develop novel composite and nanocomposite formulations, incorporating additives like nanocellulose, chitin, or clay, to enhance barrier properties and thermal stability [285]. In this context, bacterial nanocellulose (BNC) represents a distinct class of nanocellulose with increasing relevance in the field of food packaging. Unlike cellulose derived from plant sources, BNC is biosynthesized by microorganisms such as *Komagataeibacter xylinus*, producing a material characterized by exceptional purity, a highly entangled three-dimensional nanofiber network, and outstanding mechanical strength. Notably, agricultural residues can be enzymatically hydrolyzed to provide low-cost carbon sources for bacterial fermentation, thereby integrating BNC production into biowaste valorization strategies. Its intrinsic properties, such as high moisture retention, optical clarity, and biocompatibility, make BNC particularly attractive for applications in edible packaging films and functional packaging systems that enhance food preservation [286]. These innovations, combined with favorable regulatory policies and growing consumer demand for sustainable packaging, are expected to drive the commercial viability and global market integration of PHA and PLA in the future. A summary of the biopolymers used in packaging, including their origin, key characteristics, and benefits or limitations, is presented in Table 3.

## 3. Functional Properties of Agricultural Waste-Derived Biopolymers

Biopolymers synthesized from agricultural residues offer multifunctional properties that are crucial for their application in sustainable food packaging. These properties not only determine their processability and shelf-life performance but also define their environmental impact. By harnessing the molecular composition of plant-based raw materials such as cellulose, starch, lignin, and proteins, and enhancing them through physical and chemical modifications, these biopolymers can be tailored to meet specific functional requirements. This section discusses the key functional characteristics, including mechanical and barrier properties, biodegradability and compostability, as well as antimicrobial and antioxidant activities, all of which contribute to their growing potential as alternatives to petroleum-based plastics.

### 3.1. Mechanical and Barrier Properties

Mechanical strength and barrier performance are essential attributes for any food packaging material, as they determine protection against physical damage and environmental factors such as moisture, oxygen, and grease [287]. Agricultural waste-derived biopolymers, in their native form, often exhibit limited mechanical strength and poor barrier properties due to their hydrophilic nature and amorphous regions [288]. However, significant improvements can be achieved through reinforcement with nanomaterials such as nanocellulose, cellulose nanofibers (CNFs), and lignin nanoparticles. These nanoscale reinforcements provide a high aspect ratio and surface area, facilitating strong intermolecular interactions and hydrogen bonding within the polymer matrix, which increases tensile strength, elongation at break, and flexibility [289]. Additionally, cross-linking techniques using agents like citric acid or genipin create covalent bonds between polymer chains, reducing free volume and enhancing structural rigidity. Polymer blending with more hydrophobic or crystalline biopolymers further improves moisture resistance and reduces gas permeability [290]. As a result, these modified biopolymers show promising barrier performance against water vapor, oxygen, and lipids, comparable to conventional synthetic polymers, which is vital for preserving the quality and safety of food products during storage and transportation [289,291]. A relevant example is the development of bioplastic films using cellulose extracted from cocoa pod husk and fiber from sugarcane bagasse, two major agricultural waste products from the chocolate and sugar industries, respectively. In a study that assessed various cellulose: fiber ratios, the film containing 75% cellulose and 25% fiber exhibited the most favorable performance, with the lowest water absorption and water vapor permeability values. These results indicate enhanced barrier properties and moisture resistance, making this formulation particularly suitable for food packaging applications [292].

### 3.2. Biodegradability

One of the most defining features of agricultural waste-derived biopolymers is their ability to biodegrade naturally in soil, water, or composting environments, thereby contributing significantly to environmental sustainability [293]. The biodegradation rate depends on the polymer’s chemical structure, crystallinity, degree of hydrophobicity, and the presence of additives or cross-linkers. For instance, cellulose and starch-based films degrade more rapidly due to their easily hydrolyzed glycosidic bonds and hydrophilic nature. However, modifications such as esterification or blending with hydrophobic polymers may slow down the degradation process [294]. Similarly, protein-based films tend to break down quickly in moist environments due to their peptide backbone but require structural reinforcement for practical application [295]. A compelling example is the development of biodegradable films from taro peel starch (TPS), a starchy agricultural by-product. Films formulated with optimized TPS and glycerol concentrations showed homogeneous, transparent surfaces and biodegraded completely within 5 days in both simulated river water and composting soil environments. The study confirmed TPS film’s excellent degradation behavior, further supported by its amorphous structure and thermal profile, making it a promising eco-friendly material for short-lifecycle food packaging applications [296]. Compostability, defined as the ability to break down into non-toxic, stable humus under controlled composting conditions, also varies depending on processing conditions, particle size, and microbial activity. Properly designed biopolymer films can achieve complete mineralization within weeks under industrial composting settings, thus offering a closed-loop solution to food packaging waste. This property aligns with circular economy principles and makes such biopolymers attractive candidates for short-lifecycle packaging, especially in single-use applications [297,298].

### 3.3. Antimicrobial and Antioxidant Properties

Another critical functionality of biopolymers in food packaging is their ability to extend shelf life through antimicrobial and antioxidant activities. Agricultural waste-derived biopolymers can act as carriers for active compounds such as essential oils (e.g., oregano, thyme), plant-derived polyphenols, and metallic nanoparticles (e.g., silver, zinc oxide), which possess strong bioactive properties [299]. These additives can be incorporated into the polymer matrix or applied as surface coatings, enabling controlled release of antimicrobial agents in response to environmental conditions such as humidity or pH. For example, incorporating clove or cinnamon oil into cellulose films provides inhibitory effects against Escherichia coli and Staphylococcus aureus, while phenolic compounds from grape pomace or green tea exhibit antioxidant behavior by scavenging free radicals [300]. A recent study demonstrated that cellulose extracted from *Robinia pseudoacacia* (black locust) pods, an invasive plant species considered a form of biomass waste, can be crosslinked with citric acid and incorporated into PVA films, then functionalized with ferulic acid (FA). These films exhibited strong antioxidant profiles (measured via CUPRAC, FRAP, and TEAC methods) and antimicrobial activity against bacteria, yeasts, and molds, with particular efficacy against Gram-positive bacteria and yeasts. The swelling degree and bioactive release were tunable through FA content, suggesting suitability for packaging food products with water activity <0.95. This highlights the dual environmental and functional *benefits* of repurposing invasive biomass for active food packaging [301]. Furthermore, biopolymer-based edible coatings enriched with such agents can be directly applied onto fruits, vegetables, and meats to reduce microbial spoilage without altering sensory qualities. This approach not only enhances food safety but also reduces reliance on synthetic preservatives and plastic wraps, offering a clean-label and eco-friendly packaging alternative. The synergy between biodegradable materials and active compounds positions these systems as innovative solutions in the domain of active and intelligent food packaging [302].

## 4. Challenges and Limitations

Despite their numerous environmental and functional advantages, agricultural waste-derived biopolymers face several obstacles that limit their widespread industrial application. One of the primary challenges lies in scaling up production [303]. Although laboratory-scale synthesis of biopolymers from agricultural residues has shown promising results, transitioning to large-scale manufacturing remains difficult due to high processing costs, energy demands, and the need for specialized equipment. Extraction processes often require multiple steps, including pretreatment, hydrolysis, purification, and chemical modification, all of which contribute to increased operational complexity and economic burden [304].

Another significant barrier is related to performance limitations. A major trade-off exists between biodegradability and the mechanical or barrier properties essential for food packaging [305]. For instance, highly biodegradable films tend to be hydrophilic, resulting in poor water resistance and reduced durability, especially in humid environments. Additionally, the brittleness and lower tensile strength of some starch- or protein-based films restrict their use in high-performance packaging applications. Although reinforcement with nanofillers and blending with other polymers can mitigate these weaknesses, such enhancements often lead to increased costs or compromise in compostability, thus affecting the material’s overall sustainability [306].

Regulatory compliance and consumer perception also pose significant hurdles. For biopolymers to be used in direct food contact, they must adhere to strict food safety standards and migration limits established by regulatory bodies such as the Food and Drug Administration (FDA) or European Food Safety Authority (EFSA). Meeting these requirements can be time-consuming and costly, particularly when novel additives or processing aids are used. Moreover, consumer acceptance is influenced by aesthetic appeal, functional performance, labeling clarity, and price competitiveness compared to conventional plastic packaging. The lack of awareness or misinterpretation of biodegradable labels can lead to improper disposal, reducing the environmental benefits intended by these materials [307,308].

## 5. Future Prospects and Sustainability Outlook

**Figure 11:**
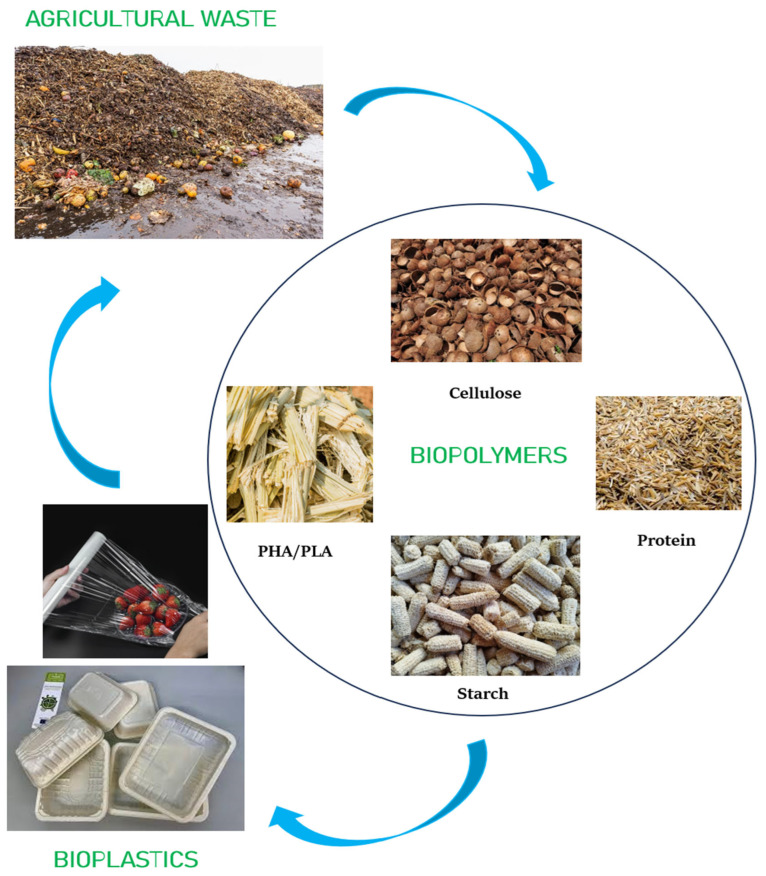
Flowchart illustrating the transformation of agricultural waste into biopolymers for food packaging.

Despite the challenges, the future of agricultural waste-derived biopolymers is highly promising, especially as industries and governments shift toward more sustainable practices, especially food packaging industries. Agricultural residues such as rice husks, wheat straw, and fruit peels undergo pre-treatment to remove impurities, followed by the extraction of biopolymers like cellulose, starch, or protein using eco-friendly methods. These extracted compounds are then purified and, if necessary, chemically modified to improve their functional properties. The modified biopolymers are processed into films or composites using techniques such as casting or extrusion. Finally, the resulting biopolymer-based materials are fabricated into packaging products that are biodegradable, sustainable, and suitable for food applications as depicted in Figure 11. A key direction is the integration of these biopolymers into circular economy models, where agricultural residues, once considered waste, are upcycled into value-added products [309]. This not only contributes to waste minimization and resource efficiency but also supports the creation of closed-loop systems, where biopolymer packaging can eventually biodegrade and return nutrients to the soil. Such an approach is aligned with global zero-waste initiatives and Sustainable Development Goals (SDGs), particularly in Goal 12 (Responsible Consumption and Production) and Goal 13 (Climate Action) [310].

To further unlock the potential of these materials, ongoing research is directed toward developing cost-effective, multifunctional, and scalable solutions. Innovations in biotechnology and genetic engineering are enabling the tailoring of microbial strains for higher biopolymer yield, reduced fermentation time, and utilization of diverse low-cost substrates [284]. Nanotechnology is also playing a transformative role, where nano-enhanced biocomposites are engineered for superior strength, thermal stability, and barrier properties. Additionally, hybrid composite systems that combine different types of biopolymers or integrate inorganic fillers are being investigated to achieve a balance between biodegradability and durability without compromising environmental performance [311].

In summary, while the journey toward mainstream adoption of agricultural waste-derived biopolymers presents multiple scientific and technical challenges, continued interdisciplinary collaboration between researchers, industries, and policymakers is key to accelerating progress. With appropriate investments in infrastructure, innovation, and education, these materials can play a central role in shaping a sustainable, bio-based economy.

## Figures and Tables

**Table 1:** Biopolymer-rich agricultural residues and their composition.

Biopolymer	Sources	Extraction Method	Yield (%)	References
**Cellulose**	Rice Husk	Sodium hypochlorite solution followed by ultrasonication	34	[32]
	Corn cobs	Autohydrolysis for hemicellulose removal followed by alkaline delignification	77	[33]
**Starch**	Litchi seed	0.16% sodium bisulfite solution	21.4	[34]
	Mango kernel	UAE in 1% sodium bisulfite solution	54	[35]
**PHA**	Mixture of molasses and olive oil	Acid pretreatment	2.03 g/L final concentration of PHA	[36]
**PLA**	Grape stalks	Steam explosion	0.98 g/g Glu	[37]

**Table 2:** Yields of nanocrystalline cellulose (NCC) obtained from pre-isolated cellulose derived from various agricultural wastes via acid hydrolysis. Yield values are based on the mass of purified cellulose, not the original raw material, unless otherwise specified.

Agricultural Waste	Extraction Method	Yield (%)	References
Bamboo fiber	64% sulfuric acid at 45 °C for 45 min	22	[84]
Lemon seeds	64% sulfuric acid solution at 45 °C for 1.5 h	27.61	[85]
Khat waste	64% sulfuric acid at 45 °C for 60 min	49	[86]
Sugarcane straw	55% sulfuric acid at 50 °C for 15 min	21.8	[87]
Garlic stalk	58% sulfuric acid at 55 ± 1 °C for 120 min	41.98	[88]
	33% hydrochloric acid at 60 ± 1 °C for 240 min	50.56	

**Table 3:** Summary of biopolymers used in food packaging applications.

Biopolymer	Sources	Properties	Advantages	Disadvantages
**Cellulose**	Agricultural residues such as rice husk, wheat straw, corn stalks, cotton linters, wood pulp waste	Good mechanical strength, moderate barrier to gases, biodegradable, water-sensitive	Renewable, compostable, widely available, and form transparent films	Poor moisture barrier, requires modification for water resistance
**Starch**	Potato peel, corn husk, cassava peels	Good oxygen barrier, poor water resistance, brittle without plasticizers, biodegradable	Cheap, abundant, compostable, edible	Poor moisture barrier, brittle, needs additives for flexibility
**Soy Protein**	Soybean meal (byproduct of soybean oil extraction)	Good oxygen barrier, moderate mechanical properties, water-sensitive, biodegradable	Renewable, forms flexible films, good barrier to gases	Poor water resistance, allergen potential, and limited mechanical strength
**Whey Protein**	Whey (byproduct of cheese and casein production)	Excellent oxygen barrier, transparent films, water-sensitive, biodegradable	Utilizes dairy waste, good clarity, and renewable	Hygroscopic, poor water barrier, brittle without plasticizers
**Casein**	Skimmed milk or curdled milk	Good oxygen barrier, smooth films, biodegradable	Renewable, good film-forming ability	Sensitive to moisture, allergenic, and limited mechanical strength
**Gelatin**	Slaughterhouse waste (bones, hides, and connective tissues)	Good mechanical strength and flexibility, water-soluble, and biodegradable	Flexible, transparent, good gas barrier	Derived from animals, sensitive to humidity, water-soluble
**Gluten**	Wheat gluten (byproduct of wheat starch or flour processing)	Good gas barrier, biodegradable, water-sensitive	Renewable, forms cohesive films	Allergen, poor water resistance, and limited commercial availability
**PHA (Polyhydroxyalkanoates)**	Fermentation of waste oils and agro-industrial effluents	Good mechanical strength, water-insoluble, biodegradable in marine and soil environments	Fully biodegradable, compostable, good barrier properties	Expensive, limited industrial production
**PLA (Polylactic Acid)**	Fermentation of sugarcane bagasse, corn stalks, and sugar beet pulp	High transparency, good strength, brittle, biodegradable in industrial compost	Biodegradable, clear, good printability, and food-safe	Brittle, low thermal resistance, and it requires industrial composting

## Data Availability

Not applicable.
